# Integrative bioengineering strategies for endometrial regeneration: From biomaterials and stem cells to organoids and organ-on-a-chip technologies

**DOI:** 10.7150/thno.123298

**Published:** 2026-01-01

**Authors:** Soo-Rim Kim, Hwa-Yong Lee

**Affiliations:** 1 Department of Health Sciences and Technology, GAIHST, Gachon University, Incheon 21999, Republic of Korea.; 2 Department of Molecular Medicine, School of Medicine, Gachon University, Incheon 406-840, Republic of Korea.; 3 Division of Science Education, Kangwon National University, Chuncheon 24341, Republic of Korea.

**Keywords:** endometrial regeneration, tissue engineering, biomaterials, stem cells, 3D bioprinting, organoids, organ-on-a-chip, infertility

## Abstract

Endometrial regeneration remains a significant clinical challenge for women with intrauterine adhesions (IUAs), thin endometrium, or uterine factor infertility, conditions that severely impair fertility and reproductive outcomes. Traditional hormonal and surgical interventions often fail to restore the structural and functional integrity of damaged endometrial tissue. This review comprehensively examines integrative bioengineering strategies for endometrial regeneration, focusing on the synergistic applications of biomaterials, stem cells, organoids, and organ-on-a-chip technologies. Natural polymers such as collagen, gelatin, alginate, hyaluronic acid, and synthetic polymers including PCL, PLA, PGA, and PLGA have been comprehensively evaluated for their ability to mimic extracellular matrix, support cell proliferation, angiogenesis, and modulate immune responses. The incorporation of mesenchymal stem cells, extracellular vesicles, and growth factors into bioengineered scaffolds, such as hydrogels and nanofiber membranes, enhances regenerative efficacy. Furthermore, emerging platforms, such as endometrial organoids, 3D bioprinting, and organ-on-a-chip systems, offer physiologically relevant models for precision regenerative medicine. Innovations such as AI-assisted monitoring, 4D printing, and advanced drug delivery systems represent transformative approaches to overcome current therapeutic limitations. This review highlights the convergence of materials science, stem cell biology, and microengineering as a foundation for next-generation, personalized therapies aimed at restoring endometrial function and fertility. In addition, the review highlights biomaterial-based strategies as the foundation of endometrial regeneration, by detailing how natural polymers (e.g., collagen, gelatin, alginate, hyaluronic acid) and synthetic polymers (e.g., PCL, PLA, PLGA) support tissue repair structurally and by mediating biological functions. The integration of advanced technologies, such as 4D printing, AI-assisted monitoring, and stem cell-derived extracellular vesicle delivery has emerged as a transformative direction for overcoming current clinical challenges. Collectively, these approaches offer a next-generation therapeutic paradigm for restoring endometrial function and fertility.

## 1. Introduction

The endometrium, a dynamic mucosal tissue, undergoes cyclical regeneration, differentiation, and shedding in response to hormonal fluctuations throughout the menstrual cycle. Hormones regulate the growth and secretory activities of the endometrium to ensure that the uterus is adequately prepared for pregnancy 1, 2. Following ovulation, the fertilized egg travels through the fallopian tube to the endometrium for implantation, initiating further development 3. However, the endometrium is also highly susceptible to injury, inflammation, and pathological remodeling, which can lead to IUAs, thin endometrium, endometrial fibrosis, or atrophy. These conditions significantly contribute to infertility, recurrent implantation failure, and pregnancy loss, and remain clinically challenging to manage using conventional hormonal or surgical therapies 4, 5. Notably, injury to the basal layer of the endometrium, which harbors the stem/progenitor cell niches essential for cyclical regeneration 6, represents the fundamental pathological basis of IUAs 7, thin endometrium 8, and fibrosis 9. Once the basal layer is disrupted, the endometrium loses its intrinsic capacity for self-renewal, resulting in irreversible scarring, impaired receptivity, and infertility 10. This central pathological mechanism underscores the urgent clinical need for regenerative strategies that are able to reconstruct both the structural and functional integrity of the basal layer.

Regenerative medicine and tissue engineering present promising strategies for restoring damaged or diseased tissues, including reproductive organs, through the use of biocompatible scaffolds that facilitate cell growth and tissue regeneration 11. These technologies have been successfully applied to a variety of tissues, such as bone 12, cartilage 13, and liver 14, and are now gaining traction in the field of female reproductive health 15, 16. The regeneration of female reproductive tissues poses significant challenges due to their intricate biological and functional properties 1, 17. In recent years, tissue engineering and regenerative medicine have become pivotal for restoring endometrial function and addressing uterine factor infertility. The creation of bioengineered scaffolds that replicate the structural and biochemical characteristics of the native endometrial extracellular matrix (eECM) is central to these strategies. These scaffolds provide mechanical support and guide cellular proliferation, migration, and differentiation 18, 19. They must also naturally degrade within the body to yield by-products that are safely metabolized 20, 21. Both natural and synthetic polymers are being investigated in this context.

Synthetic polymers such as poly(ε-caprolactone) (PCL) 22, polyglycolic acid (PGA) 23, and poly(lactic acid) (PLA) 24 permit precise control of physical and chemical properties, though they require careful modification to mimic the biological functions of native tissues 25. Conversely, natural materials such as collagen 26, gelatin 27, and alginate 28 exhibit excellent biocompatibility but are confronted by challenges, including potential immune responses 21 and quality variability 29. Recent advancements, such as the deployment of decellularized extracellular matrices (dECMs) and hydrogels, have produced more effective scaffolds that closely mimic natural ECM and facilitate the regeneration of complex reproductive tissues like endometrium 30, ovaries 31, and fallopian tubes 32.

The integration of stem cells, particularly mesenchymal stem cells (MSCs), derived from sources like the umbilical cord, bone marrow, placenta, or the endometrium itself, into biofunctional scaffolds is a pivotal innovation in this area 33-35. These cells contribute directly to tissue regeneration through differentiation and matrix deposition and exert significant paracrine effects by releasing cytokines, growth factors, and extracellular vesicles, such as exosomes 36, 37. Interestingly, the incorporation of MSC-derived exosomes into injectable or thermosensitive hydrogels has been demonstrated to promote angiogenesis, re-epithelialization, immunomodulation, and to restore endometrial receptivity in various preclinical models 38. These composite platforms offer a cell-free, low-immunogenicity alternative to traditional stem cell therapies and are being actively investigated for clinical applications.

Concurrently, bioengineering efforts have produced advanced *in vitro* models, including endometrial organoids 39, 40 and organ-on-a-chip systems 41, 42, that effectively mimic the 3D architecture, hormone sensitivity, and immune milieu of the endometrium with exceptional fidelity. Endometrial organoids, derived from primary epithelial or stromal cells, are now cultivated in synthetic or decellularized ECM-based hydrogels to probe molecular mechanisms of regeneration, disease development, and drug responsiveness 43. Moreover, vascularized endometrium-on-a-chip platforms incorporating epithelial, stromal, endothelial, and immune cells enable real-time monitoring of tissue remodeling and endocrine signaling within physiologically relevant microfluidic settings 44. These systems are expanding our ability to investigate complex reproductive phenomena *in vitro* and show potential as diagnostic tools for tailored regenerative therapies.

In addition to these advancements, 3D bioprinting technologies are enabling the fabrication of artificial endometrial tissues with high spatial precision and functional complexity 45, 46. By utilizing bioinks composed of natural and synthetic polymers, often combined with hormones, cytokines, or stem cells, researchers have successfully engineered multi-layered structures that resemble native endometrial basal, stromal, and luminal layers 46-48. These platforms exhibit hormone responsiveness, vascularization, and successful embryo implantation in preclinical models, and are being actively refined for transplantation and intrauterine repair applications.

This review provides a comprehensive overview of recent advancements in the development and application of natural and synthetic biomaterials for endometrial regeneration, with particular focus on stem cell integration, organoid and organ-on-chip modeling, and biofabrication strategies. It critically evaluates the biological performance, engineering design, and translational potential of each approach, while highlighting emerging technologies that could accelerate the development of clinically viable therapies for endometrial injury, infertility, and uterine reconstruction. By integrating multidisciplinary innovations across materials science, reproductive biology, and biomedical engineering, this field is rapidly advancing towards personalized, minimally invasive, and highly effective regenerative treatments for reproductive medicine.

## 2. Biomaterials for endometrial tissue engineering

### 2.1. Natural biomaterials for endometrial regeneration

#### 2.1.1. Collagen and endometrial regeneration

Collagen, the predominant structural protein in the ECM and a biocompatible, medically approved scaffold, has become essential in endometrial tissue engineering due to its superior biocompatibility, low immunogenicity, and inherent bioactivity 49. Its role in maintaining tissue structure and facilitating cell-matrix interactions renders it ideal for replicating the dynamic and complex microenvironment of the endometrium, which experiences significant remodeling during the menstrual cycle 50. Furthermore, recent developments have shown that collagen-based scaffolds actively influence cellular behaviors, such as adhesion, migration, differentiation, and paracrine signaling, while also providing vital mechanical support 51. Collagen plays a crucial role in mediating communication between cells and their surrounding extracellular scaffold 52. Within the endometrium, type III collagen (COL III) is particularly abundant, and represents a significant structural component of the extracellular framework 53.

Gao et al. established that collagen significantly boosted the regenerative potential of human umbilical cord mesenchymal stem cells (hUCMSCs) in a rat model of intrauterine adhesion (IUA). The application of a hUCMSC-laden collagen scaffold substantially enhanced endometrial thickness, glandular density, and vascularization, while diminishing fibrotic deposition, compared to hUCMCs or collagen alone 54. This combined approach also further improved the expression of crucial endometrial receptivity markers, such as estrogen receptor (ER) α, progesterone receptor (PR), vascular endothelial growth factor (VEGF), leukemia inhibitory factor (LIF), insulin-like growth factor 1 (IGF-1), and Integrin β3, and restored fertility to near-normal levels. These results emphasize the dual functionality of collagen as a structural framework and a bioactive matrix that enhances stem cell-driven endometrial regeneration, underscoring its potential to treat uterine infertility.

Kendirci-Katirci et al. devised a 3D endometrium-like culture system to explore the intricacies of embryo implantation and highlighted the crucial roles played by collagen-based scaffolds in supporting cellular activities and replicating the endometrial microenvironment. Utilizing endometrial epithelial (RL95-2) and trophoblast-like (JAR) cell lines, they evaluated collagen foam (COL/FO), collagen fiber (COL/FI), and bacterial cellulose scaffolds. COL/FO and COL/FI scaffolds were found to facilitate spheroid formation, invasion, and the mesenchymal transition of trophoblast cells, as indicated by increased levels of EMT markers (N-cadherin, vimentin, α-SMA, and Syndecan-1) and diminished E-cadherin levels 55. Notably, the COL/FI scaffolds preserved cellular topography, while the COL/FO scaffolds more effectively supported invasive behavior. These findings highlight the versatility of collagen as a biomaterial that enhances cell proliferation and structural organization, enabling dynamic processes crucial for endometrial regeneration.

**Figure 1:**
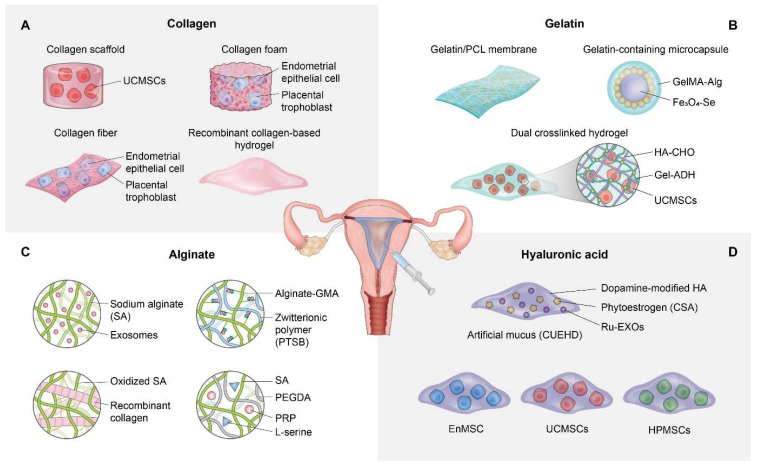
** Natural biomaterial-based strategies for endometrial regeneration.** Collagen scaffolds provide biomimetic structural support and bioactive cues that enhance cell adhesion, proliferation, and paracrine signaling. When combined with hUCMSCs, collagen matrices promote epithelial regeneration, angiogenesis, and reduce fibrosis in IUA models. Additionally, collagen foams and fibers support dynamic trophoblast invasion and epithelial-mesenchymal transition, while recombinant type III collagen (rCol III)-based hydrogels facilitate anti-fibrotic effects and functional endometrial regeneration (A). Gelatin and its derivatives (e.g., GelMA) offer tunable, biodegradable scaffolds enriched with cell adhesion motifs. Electrospun gelatin/PCL membranes restore endometrial thickness and glandular structure while modulating inflammation and enhancing vascularization. Gelatin-containing microcapsules and injectable hydrogels enable responsive drug delivery and sustained stem cell retention, facilitating immunomodulation, re-epithelialization, and improved fertility outcomes (B). Alginate hydrogels, often combined with bioactive components such as exosomes or collagen, serve as injectable, shape-conforming scaffolds for intrauterine repair. These materials support mesenchymal-to-epithelial transition, reduce fibrotic remodeling, and enhance endometrial receptivity. Alginate composites have also been shown to restore hormonal signaling pathways and support successful embryo implantation in IUA models (C). Hyaluronic acid (HA) scaffolds are leveraged for their high biocompatibility, viscoelasticity, and ability to support moisture retention and cell migration. Modified HA hydrogels deliver stem cells, phytoestrogens, or bioactive drugs, promoting endometrial regeneration via anti-inflammatory, pro-angiogenic, and anti-fibrotic mechanisms. These strategies restore hormonal receptor expression, stimulate vascularization, and improve pregnancy outcomes in preclinical uterine injury models (D).

Wang et al. engineered a temperature-responsive hydrogel system that incorporated recombinant type III collagen (rCol III), to prevent intrauterine adhesion (IUA) while enhancing functional endometrial regeneration. Leveraging the biocompatibility and bioactivity of rCol III, the hydrogel facilitated prolonged retention at sites of uterine injury, while also enabling sustained release and effective modulation of the regenerative microenvironment 56. *In vitro*, rCol III hydrogels enhanced endometrial stromal cell migration, angiogenesis, and reduced TGF-β1 expression, a critical pro-fibrotic mediator. On the other hand, *in vivo* studies utilizing a rat IUA model revealed that 1 wt% rCol III hydrogels significantly improved glandular regeneration, restored endometrial thickness, reduced fibrosis, and decreased inflammatory cytokine levels, compared to control or hyaluronic acid-based treatments. These findings highlight the translational potential of collagen-integrated biomaterials as effective anti-adhesive and pro-regenerative platforms for uterine repair. Figure 1A presents a comprehensive schematic illustrating the application of collagen-based strategies for endometrial regeneration.

Although collagen-based scaffolds consistently demonstrate favorable outcomes in preclinical studies, critical evaluation reveals several inconsistencies and unresolved challenges. For example, while Gao et al. reported enhanced glandular density and fertility restoration with hUCMSC-collagen constructs, other studies using collagen foams or fibers showed divergent effects on trophoblast invasion, with COL/FO promoting aggressive mesenchymal transition, whereas COL/FI better preserved physiological topography. These differences underscore how scaffold architecture, porosity, and processing methods may critically alter cell-matrix interactions and downstream signaling; nevertheless, direct head-to-head comparisons are scarce. Furthermore, while recombinant type III collagen hydrogels suppressed TGF-β1 and reduced fibrosis in animal models, their long-term stability, degradation kinetics, and reproducibility across different injury severities remain inadequately studied. A further methodological limitation is the reliance on rodent IUA models, which only partially recapitulate the cyclic hormonal remodeling and immune complexity of the human endometrium, raising concerns about translational fidelity. Clinically, variability in collagen source (xenogenic *vs.* recombinant), batch-to-batch consistency, and potential immunogenicity pose additional barriers. Importantly, the dual role of collagen in either promoting regeneration or driving fibrosis—depending on the microenvironmental context—remains insufficiently understood, with conflicting evidence regarding its influence on EMT and fibrotic cascades. Collectively, these issues highlight that while collagen scaffolds represent a promising bioactive platform, further comparative, mechanistic, and translational studies are essential to determine their optimal design parameters and clinical applicability.

Beyond serving as a structural scaffold, collagen actively interacts with uterine cells to regulate their behavior. Increasing evidence indicates that its biological impact is mediated through intracellular signaling cascades that control matrix remodeling and tissue repair. Building on this, collagen, particularly type IV, modulates key signaling pathways in uterine cells beyond its structural role. In endometrial epithelial cells, collagen engages focal adhesion kinase (FAK) and Src signaling, which in turn regulate the activity of matrix metalloproteinases (MMP-2, MMP-9). This pathway governs collagen hydrolysis, cell adhesion, and migration. Inhibition of FAK downregulates Src and MMPs, leading to COL-IV accumulation, whereas FAK activation enhances MMP expression and accelerates collagen turnover 57. In addition, recombinant humanized type III collagen (rhCol III) directly modulates endometrial cell signaling beyond structural support. In macrophages, rhCol III shifts polarization from pro-inflammatory M1 to anti-inflammatory M2 phenotype, downregulating IL-6 and TLR4, while upregulating IL-10. In endometrial stromal cells, rhCol III enhances decidualization by increasing IGFBP-1 and PRL expression, and promotes ECM remodeling through upregulation of collagen I/III. Mechanistically, collagen engages DDR1/DDR2 receptors and integrin-mediated pathways, while concurrently suppressing NF-κB and YAP signaling cascades, thereby reducing inflammation and restoring regenerative capacity 58. Collectively, these findings demonstrate that collagen functions as a dynamic regulator of endometrial regeneration by orchestrating immune modulation, ECM remodeling, and intracellular signaling.

#### 2.1.2. Gelatin and endometrial regeneration

Gelatin, a denatured form of collagen, has garnered increasing attention in the endometrial tissue engineering field due to its excellent biocompatibility, biodegradability, and chemical modifiability 59. Collagen provides structural support and offers abundant cell adhesion motifs and modifiable functional groups, making it ideal for the fabrication of hydrogels, electrospun membranes, and bioactive delivery systems 60. Recent advances have shown that gelatin-based scaffolds, particularly when combined with polymers or responsive elements, actively enhance re-epithelialization, stromal regeneration, angiogenesis, and immune modulation, while serving as physical barriers that prevent intrauterine adhesion 61. Additionally, when integrated with stem cells or therapeutic agents, gelatin matrices have demonstrated enhanced capacity to restore endometrial receptivity and fertility 62.

Wang et al. explored the regenerative potential of electrospun gelatin/polycaprolactone (GT/PCL) membranes in a rat model of endometrial injury and demonstrated their effectiveness at restoring endometrial architecture and fertility. These GT/PCL membranes displayed favorable porosity, biocompatibility, and mechanical properties, enabling them to act as physical barriers that prevent IUA while promoting tissue regeneration. Histologic and molecular analyses indicated that GT/PCL implantation significantly increased endometrial thickness, re-epithelialization (as indicated by CK19), stromal cell proliferation (as indicated by vimentin), neovascularization (as indicated by CD34 and VEGF), and estrogen receptor expression, while reducing collagen deposition and TNF (an inflammatory cytokine) levels 63. Notably, uterine morphology normalized, and improved embryo implantation rates were observed in treatment groups, confirming functional recovery. These findings highlight the versatility of gelatin as a natural biomaterial scaffold when incorporated into composite membranes for endometrial regeneration and anti-adhesion applications.

Wang et al. developed multifunctional microcapsules (A/G-Fe₃O₄-Se) with a dual-network hydrogel shell comprised of alginate and gelatin methacryloyl (GelMA), which encapsulated magnetic selenium-coated Fe₃O₄ nanoparticles and an ultrasound-sensitive decafluoropentane core. Utilizing microfluidic technology, these microcapsules enhanced biocompatibility, magnetic responsiveness, and shape adaptability, the latter facilitated coverage of the irregular uterine cavity and effectively preventing intrauterine adhesion (IUA) 64. *In vitro*, the microcapsules demonstrated antioxidant, antibacterial, and regenerative properties, while *in vivo* application in a rat IUA model decreased oxidative stress, enhanced endometrial restoration, and significantly upregulated markers of endometrial receptivity and fertility outcomes.

Zhang et al. engineered an injectable hydrogel using oxidized hyaluronic acid (HA-CHO) and hydrazide-grafted gelatin (Gel-ADH) to deliver human umbilical cord mesenchymal stem cells (hUCMSCs) for endometrial regeneration. This HA/Gel hydrogel exhibited excellent biocompatibility, self-healing properties, and sustained hUCMSC retention at sites of uterine injury. In a rat model of intrauterine adhesion (IUA), the hUCMSC-loaded hydrogel significantly increased endometrial thickness, glandular and vascular density, and reduced fibrotic remodeling 65. Mechanistically, this therapy activated the MEK/ERK1/2 signaling pathway, upregulated VEGF expression, and suppressed the inflammatory microenvironment by reducing IL-1β and IL-6 levels and elevating IL-10 levels. Furthermore, these regenerative effects restored embryo implantation and successful live birth rates without adverse maternal or fetal outcomes.

Despite promising results, the application of gelatin-based scaffolds to endometrial regeneration is not without limitations and conflicting findings. While GT/PCL membranes effectively enhanced epithelial repair, angiogenesis, and fertility recovery in rat models, their performance remains highly dependent on the scaffold architecture, degradation kinetics, and polymer composition, which vary across studies, and complicate direct comparisons. Moreover, while multifunctional gelatin-based microcapsules (e.g., A/G-Fe₃O₄-Se) demonstrated antioxidant and antibacterial activity, questions remain about the long-term biosafety and reproducibility of incorporating magnetic nanoparticles and ultrasound-sensitive elements in clinical settings. Similarly, while HA/Gel hydrogels with hUCMSCs achieved significant anti-fibrotic and pro-regenerative effects through MEK/ERK1/2 activation, the reliance on xenogeneic or allogeneic stem cells introduces variability in immune response, scalability, and regulatory acceptability. Notably, divergent outcomes are observed regarding the extent of fibrosis suppression and pregnancy restoration, reflecting differences in injury severity, animal models, and follow-up periods. Methodologically, most studies remain confined to small-animal IUA models, which fail to capture the complex hormonal, vascular, and immune dynamics of the human uterus, raising concerns about translational fidelity. Furthermore, the rapid enzymatic degradation of gelatin *in vivo*, unless chemically modified, may compromise scaffold persistence and regenerative efficacy. Taken together, while gelatin and its derivatives offer versatile platforms with strong bioactivity, to define the true translational potential of gelatin-based systems in clinical endometrial repair, future work must focus on head-to-head comparative studies, standardized fabrication protocols, and large-animal validations.

Gelatin-based scaffolds actively regulate endometrial repair by modulating cell-specific signaling pathways and gene expression. In epithelial cells, they enhance CK19 expression to accelerate re-epithelialization, while in stromal cells they upregulate vimentin, supporting proliferation and ECM remodeling. Gelatin also promotes angiogenesis through increased VEGF receptor and CD34 expression, while suppressing TNF and the downstream TGF-β1/Smad3 pathway to inhibit fibrosis. Furthermore, elevated ERα expression indicates enhanced hormonal responsiveness and regenerative potential 63. Enzyme cross-linked gelatin hydrogels delivering menstrual blood-derived MSC significantly promoted endometrial repair by modulating key molecular pathways. RNA-seq analysis revealed activation of immune response-related and estrogen release-related signaling pathways, accompanied by upregulation of ORM1 (anti-fibrotic) and downregulation of H19 and HMOX1 (pro-fibrotic). These findings demonstrate that gelatin-based hydrogels provide structural support, while also actively regulating gene expression and signaling cascades that govern fibrosis, angiogenesis, and hormonal responsiveness in the injured endometrium 66. Collectively, these findings highlight that gelatin-based biomaterials function both as structural scaffolds, and as active regulators of signaling pathways and gene expression in the endometrium. By orchestrating epithelial repair, stromal remodeling, angiogenesis, anti-fibrotic signaling, and hormonal responsiveness, gelatin scaffolds provide a mechanistic foundation for their therapeutic potential in endometrial regeneration.

#### 2.1.3. Alginate and endometrial regeneration

Alginate, a naturally derived polysaccharide extracted from brown seaweed, has established itself as a highly versatile biomaterial in the tissue engineering field due to its biocompatibility, injectability, tunable gelation properties, and ability to form hydrogels under mild conditions 67. The ionic cross-linking network of alginate provides a robust matrix for cell delivery, localized therapeutic retention, and controlled release of bioactive molecules 68, 69, making it an excellent candidate for promoting uterine repair. Recent advancements have further increased the functionality of alginate through chemical modifications and hybridization with bioactive compounds, resulting in dual-action scaffolds that serve as physical barriers and regenerative agents. These alginate-based platforms have been demonstrated to be effective at enhancing angiogenesis, modulating fibrosis, restoring hormonal receptor expression, and improving embryo implantation in preclinical IUA models. Notably, Liang et al. developed a novel therapeutic strategy for endometrial regeneration by encapsulating decidual stromal cell-derived exosomes (DSC-exos) within a sodium alginate hydrogel (SAH) to address IUA and restore fertility. The biofriendly, injectable alginate-based scaffold enabled sustained exosome release and retention within the uterine cavity 70. In a murine IUA model, the DSC-exos/SAH construct significantly enhanced angiogenesis, promoted mesenchymal-to-epithelial transformation (MET), remodeled collagen fibers, and improved endometrial thickness and glandular density. In addition, it increased the expression of markers associated with endometrial receptivity (PR, p-STAT3, Ki67) and enhanced embryo implantation rates. Zhang et al. engineered an injectable alginate-based zwitterionic hydrogel (Alg-GMA/PTSB) by integrating methacrylated alginate with a thiolated zwitterionic polymer to promote endometrial repair and restore fertility in cases of intrauterine adhesion (IUA). The hydrogel produced demonstrated enhanced biocompatibility, biodegradability, and antifouling properties. In a rat model of IUA, the hydrogel promoted epithelial regeneration, angiogenesis, and reduced fibrosis by suppressing the TGF-β1/Smad3 pathway 71. In addition, it restored the expression of estrogen and progesterone receptors, increased endometrial receptivity markers, and significantly improved embryo implantation and fertility outcomes.

Fang et al. developed an injectable, dual-crosslinked hydrogel composed of oxidized sodium alginate (OSA) and recombinant type III collagen (RC). This hydrogel was designed to promote rapid and sustained regeneration of damaged endometrium without requiring exogenous cells or hormones. In addition, the hydrogel is injectable, biodegradable, and structurally stable, and forms covalent and ionic crosslinks that enable it to conform to the irregular contours of the uterine cavity and maintain a prolonged therapeutic presence 72. In a chemically induced mouse model of severe endometrial injury, the OSA/RC hydrogel significantly enhanced epithelial and stromal recovery, reduced fibrosis, and restored hormonal receptor expression (ERα, PR) without inducing aberrant epithelial proliferation. Histological analysis confirmed the re-establishment of normal uterine architecture and function. This positions alginate-collagen composites as promising non-cellular, biomimetic platforms for endometrial regeneration and anti-adhesion therapy.

Xie et al. developed an injectable, biodegradable hydrogel composed of polyethylene glycol diacrylate (PEGDA), sodium alginate (SA), and L-serine. They then enhanced this platform by incorporating platelet-rich plasma (PRP) to create a multifunctional platform (PSL/PRP hydrogel) for treating IUAs. Leveraging the rapid gelation properties conferred by L-serine and the mechanical reinforcement afforded by alginate, the hydrogel demonstrated stability, injectability, and biodegradation properties suitable for endometrial applications 73. In a rat model of intrauterine adhesion, the PSL/PRP hydrogel significantly suppressed fibrotic remodeling, increased endometrial thickness and glandular density, and facilitated re-epithelialization and angiogenesis, while continuously releasing essential growth factors, such as PDGF and VEGF. This integrated approach led to the restoration of fertility and embryo implantation rates, which highlighted the effectiveness of alginate-integrated hydrogels as mechanically robust, bioactive scaffolds that serve as physical barriers and promote intrinsic endometrial regeneration and functional recovery.

Although alginate-based hydrogels demonstrate consistent promise in promoting epithelial recovery, angiogenesis, and fibrosis suppression in preclinical IUA models, critical evaluation highlights several limitations and unresolved issues. First, regenerative outcomes vary considerably, depending on the crosslinking strategy and hybrid composition: for example, zwitterionic alginate hydrogels showed potent anti-fibrotic effects through TGF-β1/Smad3 suppression, whereas OSA/RC composites emphasized structural biomimicry and hormone receptor restoration. While these differences suggest that scaffold chemistry and crosslinking density may substantially alter biological responses, direct comparative studies are lacking. Moreover, while encapsulation of bioactive agents such as DSC-derived exosomes or PRP clearly enhances regenerative efficacy, it remains unclear whether these benefits arise from the alginate scaffold itself, or the incorporated bioactive components, raising questions about the intrinsic *vs.* extrinsic contribution of alginate. Methodologically, most findings derive from rodent IUA or chemically induced endometrial injury models, which fail to fully replicate the hormonal cyclicity, vascular complexity, and immune heterogeneity of the human endometrium. This gap limits the predictive power of preclinical studies. Translationally, challenges remain regarding the reproducibility of alginate formulations, rapid *in vivo* degradation without chemical modification, and potential variability introduced by donor-derived additives, such as PRP or exosomes. Finally, the long-term safety of alginate composites in terms of chronic immune response, degradation byproducts, and pregnancy outcomes has not been systematically addressed. Collectively, while alginate-based systems hold strong potential as injectable, minimally invasive platforms for endometrial repair, rigorous head-to-head evaluations, large-animal studies, and standardized protocols are essential to define their true therapeutic window and accelerate clinical translation.

Recent findings demonstrate that alginate hydrogels provide structural support, while also actively modulating molecular signaling in endometrial stem cells. Alginate encapsulation enhanced germline differentiation by upregulating germ cell markers (DAZL, DDX4) and meiotic/oocyte-related genes (SCP3, GDF9, GDF9B) in endometrial stem cells. This indicates the activation of meiosis-specific signaling cascades within the 3D alginate niche, with the retinoic acid and BMP4 axis exerting a synergistic effect on lineage specification 74. These results underscore the role of alginate as a bioactive scaffold that regulates gene expression programs that are critical for reproductive regeneration. Collectively, these studies highlight that alginate-based hydrogels actively regulate uterine regeneration by modulating key signaling pathways and gene expression. Through suppression of the TGF-β1/Smad3 axis, restoration of hormone receptor expression, and induction of germline differentiation via RA-BMP4-mediated cascades (upregulating DAZL, DDX4, SCP3, GDF9), alginate functions as a bioactive scaffold that integrates structural support with molecular regulation to enhance endometrial repair and reproductive potential.

#### 2.1.4. Hyaluronic acid and endometrial regeneration

Hyaluronic acid (HA) is a naturally occurring glycosaminoglycan abundant in ECM and has become a prominent biomaterial in endometrial tissue engineering due to its superior biocompatibility, biodegradability, hydrophilicity, and tunable viscoelastic properties 75. The capacity of HA to retain moisture, facilitate cell adhesion, and modulate inflammation 76 renders it highly suitable for enhancing regeneration in delicate uterine tissues. HA-based hydrogels, when chemically modified or integrated with stem cells, bioactive molecules, or therapeutic agents, offer multifunctional advantages, including improved cell viability, anti-fibrotic properties, angiogenesis, and enhanced endometrial receptivity 77. Recent advancements have also enabled HA formulations to function as dynamic delivery systems that can reverse conditions such as thin endometrium and IUA while fostering embryo implantation and acting as anti-adhesive barriers 78, 79. These attributes collectively highlight the significant translational potential of HA for developing advanced therapies that restore endometrial function and fertility.

Li et al. developed a dopamine-modified HA-based artificial mucus (CUEHD) incorporating the phytoestrogen cajaninstilbene acid (CSA) and rat urinary exosomes (Ru-EXOs) to repair thin endometrium and improve fertility. This HA scaffold offers excellent elasticity, adhesion, and biocompatibility, ensuring prolonged retention and sustained release of therapeutic agents in the uterine cavity. In a rat model of thin endometrium, CUEHD significantly improved endometrial thickness, glandular development, and vascularization. It also restored estrogen receptor expression and reduced inflammation by modulating the ER-NLRP3-IL1β and TGFβ/Smad-Wnt/β-catenin pathways 80. This strategy not only supported embryo implantation and successful pregnancies but also demonstrated an excellent biosafety profile, thus emphasizing the translational potential of HA-based hydrogels as multifunctional platforms for endometrial regeneration.

Fan et al. developed a therapeutic strategy that combined human umbilical cord-derived mesenchymal stem cells (hUC-MSCs) with auto-crosslinked HA gel to promote endometrial regeneration and treat intrauterine adhesion (IUA). This HA gel, approved by the China Food and Drug Administration, acted as a biocompatible scaffold to enhance hUC-MSC retention, viability, and regenerative potential within the uterine cavity. In a rat model of IUA, hUC-MSCs/HA-gel complex significantly increased endometrial thickness, glandular number, and vascularization, while also reducing fibrosis and collagen deposition. This approach also elevated key markers of endometrial repair and estrogen signaling (ER, VEGF, Ki67, integrin β1), and decreased the expression of pro-fibrotic MMP-9. Notably, hUC-MSCs/HA-gel complex markedly restored fertility in treated animals 81. These results emphasize the dual regenerative and anti-adhesive capabilities of HA gel as well as its synergism with hUC-MSCs at restoring endometrial structure and function, making it a promising platform for future clinical applications.

Liu et al. developed a therapeutic strategy that combined endometrium-derived mesenchymal stem cells (eMSCs) with a biocompatible HA-based hydrogel (HA-GEL). This strategy was designed to address endometrial damage associated with fertility-sparing treatment in early-stage endometrial cancer (EC). Following a comprehensive evaluation of biomaterials, HA-GEL was selected as the optimal scaffold for its low cytotoxicity and enhanced support for eMSC viability 82. *In vivo* studies utilizing subcutaneous xenograft and intrauterine injury models confirmed the safety and efficacy of the eMSCs/HA-GEL complex. These studies demonstrated no tumorigenic risk, significant improvements in endometrial thickness, gland density, and angiogenesis, and reduced fibrosis. In addition, eMSCs/HA-GEL complex and progestin combination therapy enhanced the anti-tumor effects of progestin *in vitro* and increased pregnancy rates in animals.

Hu et al. developed an injectable, thermosensitive hydrogel composed of aldehyde-functionalized Pluronic F127 and adipic dihydrazide-modified HA (AHA). This hydrogel was designed to deliver human umbilical cord mesenchymal stem cells (UCMSCs) and asiaticoside-loaded microspheres for endometrial scar repair. This composite hydrogel exhibited excellent biocompatibility, mechanical strength, and sustained asiaticoside release, thereby creating a microenvironment that promotes cell adhesion, proliferation, and angiogenesis 83. In a rat model of uterine scarring, intrauterine transplantation of the hydrogel significantly restored endometrial morphology, promoting glandular regeneration, reducing fibrosis, and enhancing vascularization. Additionally, the system modulated inflammatory responses by downregulating TGF-β1 and inducing macrophage polarization from the M1 to M2 phenotype. These results underscore the potential of HA-based hydrogels as multifunctional carriers for stem cell and drug delivery in the context of endometrial regeneration.

Lin et al. developed a synergistic therapeutic strategy for regenerating thin endometria by encapsulating human placenta-derived mesenchymal stem cells (HP-MSCs) within a photocrosslinked HA hydrogel. The aim was to enhance cell retention, proliferation, and angiogenic potential *in vivo*. The HA hydrogel served as a supportive, injectable 3D scaffold that extended HP-MSC residence within the uterine cavity and significantly increased endometrial thickness, glandular density, vascularization, and embryo implantation rates in a chemically and mechanically injured murine model 79. Mechanistically, the encapsulated HP-MSCs activated the JNK/Erk1/2-Stat3-VEGF pathway in endometrial stromal cells and the Jak2-Stat5 and c-Fos-VEGF pathways in glandular cells, promoting proliferation, migration, and revascularization. These findings highlight the translational relevance of HA hydrogels as delivery vehicles for stem cell-based therapies targeting endometrial injury and infertility. Furthermore, HA-based biomaterials have shown substantial promise in endometrial regeneration by functioning as structural scaffolds and vehicles for delivering stem cells and therapeutic agents. Applications of HA-based strategies for endometrial regeneration are illustrated in Figure 1D.

Although HA-based biomaterials have repeatedly demonstrated robust regenerative and anti-fibrotic properties in preclinical models, critical analysis reveals important limitations and unresolved questions. For example, strategies such as CUEHD artificial mucus or HA/MSC composites achieved marked improvements in epithelial recovery, vascularization, and fertility restoration; however, their reliance on combinatorial therapies (e.g., phytochemicals, exosomes, or stem cells) makes it difficult to disentangle the intrinsic regenerative contribution of HA itself from that of the incorporated bioactive agents. Moreover, while several studies reported the suppression of fibrotic signaling via TGF-β1/Smad inhibition and modulation of inflammatory cytokines, other reports highlight variability in fibrosis outcomes, suggesting that HA's effects may strongly depend on crosslinking chemistry, degradation kinetics, and the severity of injury models. Methodologically, most findings are derived from rodent IUA or chemically-induced thin endometrium models, which do not fully replicate the complex endocrine, immune, and vascular microenvironment of the human uterus, limiting translational predictability. Importantly, long-term biosafety remains insufficiently addressed: while short-term pregnancy outcomes have been promising, potential risks associated with degradation byproducts, chronic immune activation, or tumor recurrence (in fertility-preserving cancer models) are still poorly characterized. Regulatory and practical barriers also remain—commercially approved HA gels (e.g., auto-crosslinked formulations) are limited in indication, and large-scale GMP production with consistent viscoelastic properties is not yet standardized. Taken together, while HA-based hydrogels offer versatile, multifunctional platforms for endometrial repair, future studies must prioritize head-to-head comparative analyses, large-animal validation, and long-term safety and regulatory assessments to substantiate their clinical applicability.

Overall, hyaluronic acid-based biomaterials actively orchestrate endometrial regeneration by regulating diverse signaling cascades across uterine cell types. They suppress fibrotic remodeling through modulation of the ER-NLRP3-IL1β and TGF-β/Smad-Wnt/β-catenin pathways, while enhancing repair and angiogenesis via the activation of JNK/Erk1/2-Stat3-VEGF in stromal cells, and Jak2-Stat5/c-Fos-VEGF in glandular cells. These coordinated molecular effects restore hormone responsiveness, reduce fibrosis, and promote structural and functional recovery of the endometrium.

#### 2.1.5. Critical summary and outlook for natural biomaterials-based endometrial regeneration

Natural biomaterials, such as collagen, gelatin, alginate, and hyaluronic acid, have shown remarkable promise in endometrial regeneration by recapitulating key features of the native extracellular matrix, modulating immune responses, and activating diverse molecular pathways. Although collagen and gelatin are rich in adhesion motifs and provide strong biochemical cues, their batch-to-batch variability and potential immunogenicity may limit reproducibility and clinical translation. While alginate offers excellent injectability and tunable crosslinking properties, enabling versatile hydrogel systems, its lack of inherent cell-adhesion sites necessitates chemical modification or hybridization with bioactive molecules. Despite hyaluronic acid exhibiting outstanding biocompatibility and anti-fibrotic activity, its rapid degradation and mechanical weakness require stabilization strategies. When comparing these biomaterials, collagen and gelatin excel in supporting cell adhesion and hormonal responsiveness, while alginate and HA provide superior adaptability for minimally invasive, injectable therapies. Importantly, each material demonstrates distinct effects on signaling pathways: collagen engages FAK-Src-MMP and DDR/NF-κB/YAP cascades; gelatin modulates TGF-β1/Smad3 and immune-related pathways; alginate regulates TGF-β1/Smad3 and RA-BMP4-mediated germline differentiation; and HA activates ER-NLRP3-IL1β and JNK/Erk/Stat-VEGF signaling. Despite these advances, limitations remain in achieving consistent mechanical strength, long-term integration, and large-animal validation. Moving forward, the rational design of composite systems that combine the structural fidelity of protein-based scaffolds with the tunability of polysaccharide-based hydrogels may provide a balanced solution. Moreover, head-to-head comparative studies and standardized mechanistic evaluations are needed to clarify the optimal application scenarios—whether for anti-fibrosis, angiogenesis, or hormonal regulation—and to accelerate the clinical translation of natural biomaterials in reproductive medicine.

### 2.2. Synthetic biomaterials and endometrial regeneration

#### 2.2.1. Polycaprolactone and endometrial regeneration

Polycaprolactone (PCL) is a semi-crystalline FDA-approved biodegradable polyester that has attracted much attention due to its excellent mechanical properties, tunable degradation profile, and compatibility with the electrospinning of nanofibrous scaffolds 84, 85. Although inherently hydrophobic and limited in bioactivity, recent advancements in material functionalization and hybridization have markedly enhanced its regenerative capabilities 86. When engineered with bioactive agents, combined with natural polymers, or designed for targeted drug and cell delivery, PCL-based scaffolds have proven capable of restoring tissue structure, reducing fibrosis, promoting re-epithelialization, and modulating immune responses in preclinical models 87, 88. These advancements highlight the evolving role of PCL as a versatile synthetic biomaterial for supporting structural and functional uterine regeneration.

Zhou et al. developed a novel regenerative approach by integrating adipose-derived mesenchymal stem cells (ADMSCs) with electrospun nanofibrous mats composed of silk fibroin and polycaprolactone (SF/PCL). This approach was aimed at treating severe IUAs and restoring endometrial function. The SF/PCL nanofiber scaffold provided mechanical strength and a biomimetic architecture that enhanced ADMSC attachment, proliferation, and survival. In a rat IUA model, the ADMSCs-SF/PCL system significantly promoted endometrial re-epithelialization, glandular and vascular regeneration, and reversed fibrosis by inhibiting the TGF-β1/Smad signaling pathway 89. Importantly, it also improved the endometrial immune microenvironment by restoring NK cell populations and balancing Th1/Th2 response. The composite system demonstrated prolonged and superior therapeutic efficacy to treatment with estrogen alone, underscoring the potential of PCL-based nanofiber scaffolds in endometrial regeneration and immune remodeling therapies​.

Ebrahimi et al. developed an innovative guided bone regeneration (GBR) membrane composed of PCL and PVA (polyvinyl alcohol) containing various concentrations of metformin (Met) to enhance osteogenic potential. The composite membranes demonstrated improved hydrophilicity, swelling capacity, and degradation kinetics but maintained mechanical integrity. Among the formulations examined, the membrane containing 10 wt% Met exhibited optimal bioactivity and markedly upregulated osteogenesis-related gene expression *in vitro* 90. When seeded with human endometrial stem cells (hEnSCs), the PCL/PVA/10% Met scaffold promoted osteogenic differentiation and bone tissue regeneration in a rat calvarial defect model, as evidenced by histological and morphometric findings. These results underscore the potential of Met-loaded PCL/PVA membranes preconditioned with hEnSCs as a biomaterial platform for bone defect repair in GBR applications.

Hanuman et al. engineered a bioinspired scaffold for uterine tissue regeneration by electrospinning galactose-conjugated PCL nanofibers. Recognizing the hydrophobic limitations of native PCL, the team enhanced its bioactivity and cell-interactive properties by functionalizing its surface with galactose. This approach was used to emulate native ECM and promote uterine fibroblast adhesion through L-selectin-mediated interactions. The modified scaffold was produced in random and aligned fiber configurations and exhibited increased hydrophilicity and improved mechanical strength and porosity 91. *In vitro* assays employing human uterine fibroblasts, showed superior cytocompatibility, proliferation, and ECM remodeling, as indicated by the upregulations of galectin-3, versican, laminin, and collagen I, on the modified scaffolds. In addition, the subcutaneous implantation of galactose-conjugated PCL patches in Wistar rats confirmed biocompatibility and caused minimal fibrotic response. These findings highlight the potential of surface-functionalized PCL nanofibers as innovative synthetic biomaterials for endometrial and myometrial repair applications.

**Figure 2:**
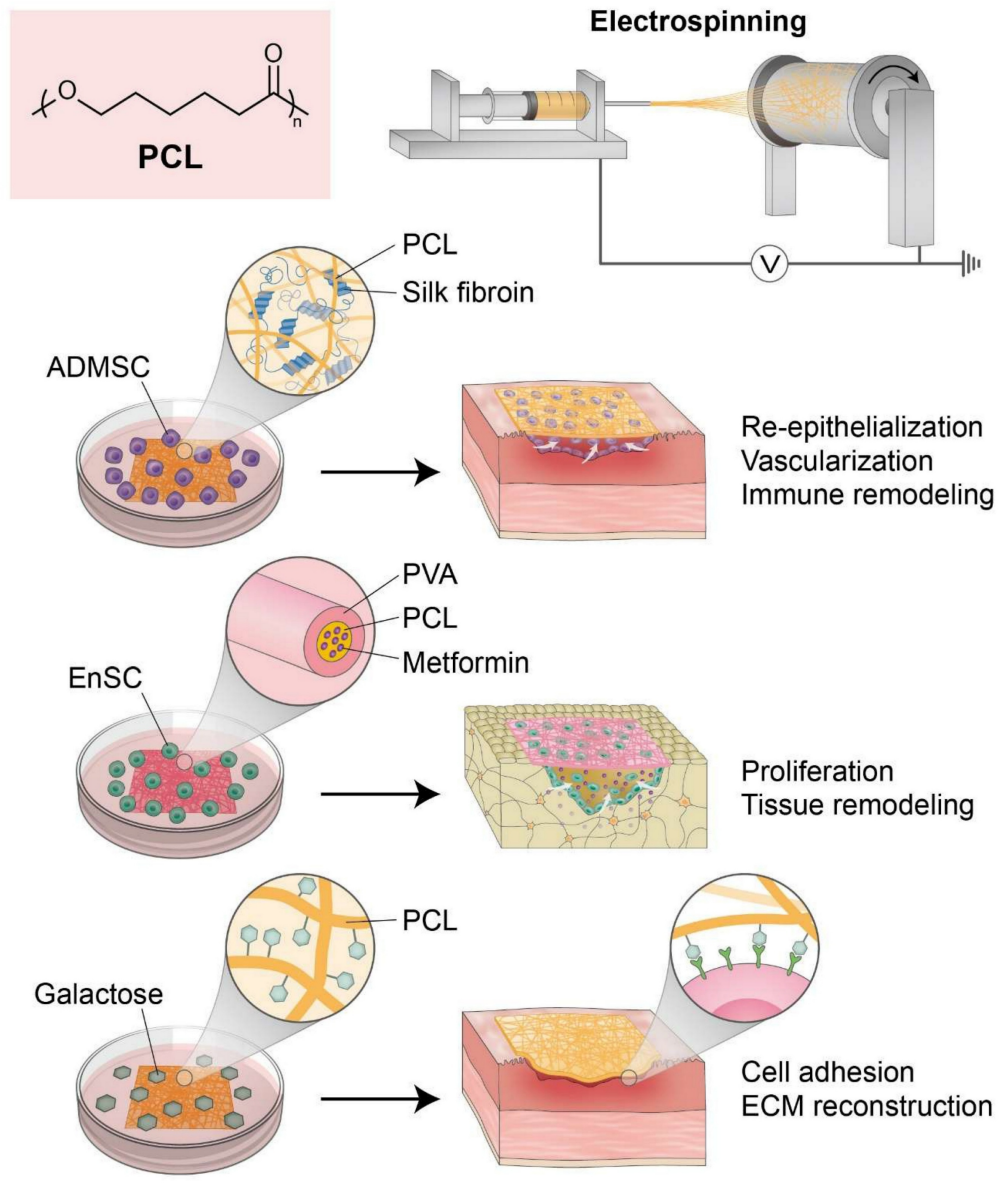
** Polycaprolactone (PCL)-based nanofibrous scaffolds for endometrial regeneration.** This figure illustrates recent advances in the design and application of PCL-based nanofibrous scaffolds for uterine tissue engineering. Due to its favorable mechanical properties and electrospinnability, PCL serves as a versatile synthetic platform, enhanced further through polymer blending, surface modification, and bioactive loading. Composite scaffolds such as silk fibroin/PCL seeded with adipose-derived mesenchymal stem cells (ADMSCs) have shown efficacy in restoring endometrial structure, reducing fibrosis, and modulating immune responses in intrauterine adhesion models. Drug-loaded PCL/PVA membranes also promote osteogenic differentiation of human endometrial stem cells (hEnSCs), broadening PCL's regenerative potential. Surface-functionalized PCL nanofibers with galactose improve hydrophilicity and support uterine fibroblast adhesion and extracellular matrix remodeling. Collectively, these PCL-based platforms offer biomimetic environments that enhance cellular integration, immune modulation, and tissue remodeling, underscoring their promise in restoring endometrial integrity and reproductive function.

Collectively, recent advances in PCL-based scaffold engineering, including fiber alignment, polymer blending, and surface modification, have substantially improved applicability for endometrial regeneration. These engineered systems exhibit enhanced cell affinity, immunomodulatory capacity, and the structural support essential for epithelial and stromal repair (Figure 2).

Despite the encouraging advances in PCL-based scaffolds, several limitations and conflicting observations warrant careful consideration. For example, ADMSC-loaded SF/PCL nanofibers demonstrated robust inhibition of the TGF-β1/Smad pathway and improved immune balance in rat IUA models, whereas galactose-functionalized PCL scaffolds primarily enhanced fibroblast adhesion and ECM remodeling *in vitro*, with more limited validation in uterine injury models. These divergent outcomes highlight how scaffold composition, surface chemistry, and fiber architecture critically shape cellular responses; however, systematic comparative studies remain scarce. Moreover, while metformin-loaded PCL/PVA scaffolds promoted osteogenic differentiation of endometrial stem cells in bone defect models, their relevance to uterine regeneration remains indirect, underscoring the need to better define tissue-specific applications. Methodologically, most studies rely on small-animal models that do not fully recapitulate the cyclic hormonal environment, immune complexity, or biomechanical forces present in the human uterus, raising questions about translational predictability. Clinically, challenges persist regarding the hydrophobic nature and slow degradation rate of PCL, which if not adequately modified, may limit host integration and lead to fibrotic encapsulation. Furthermore, regulatory acceptance of hybrid or drug-loaded PCL constructs will require stringent demonstration of long-term biosafety, reproducibility, and GMP-compliant manufacturing. Collectively, while PCL-based scaffolds represent a structurally robust and highly adaptable platform, future work must emphasize direct head-to-head comparisons of scaffold designs, long-term functional assessments in large-animal models, and standardized translational protocols to define their optimal role in endometrial repair and fertility restoration.

Recent evidence indicates that PCL-based electrospun membranes influence endometrial stem cell fate through specific gene regulatory mechanisms. While PCL supports the maintenance of mesenchymal stem cell phenotypes such as CD90 and Meflin, its composites, in particular PCL-HA, actively reshape inflammatory and regenerative signaling. H-EMSCs cultured on PCL-HA exhibit suppressed IL-6 and enhanced IL-10, VEGFA, VEGFB, and TGF-β expression, thereby reducing pro-inflammatory signaling, while promoting angiogenesis and tissue remodeling 92.

Collectively, these findings highlight that PCL-based scaffolds actively modulate endometrial regeneration by regulating both inflammatory and regenerative signaling. Through suppression of the TGF-β1/Smad pathway and transcriptional reprogramming of cytokines and growth factors (IL-6, IL-10, VEGFA, VEGFB, TGF-β), PCL and its composites orchestrate epithelial repair, angiogenesis, and fibrosis reversal, underscoring their role as bioactive regulators beyond structural support.

#### 2.2.2. Polyglycolic acid for endometrial regeneration

Polyglycolic acid (PGA), a synthetic, biodegradable aliphatic polyester, has emerged as a compelling candidate for tissue engineering due to its excellent mechanical strength, biocompatibility, and versatility in electrospinning applications 93. Its fibrous architecture closely resembles that of native ECM and promotes cellular adhesion, proliferation, and differentiation 94, 95. Engineered PGA scaffolds, particularly when seeded with endometrial or myometrial cells, have demonstrated the ability to support epithelial stratification, ECM deposition, and even live births in preclinical models. These results establish PGA as a structurally and biologically favorable platform for *in vitro* endometrial modeling and *in vivo* regenerative therapies and offer the prospect of treating uterine factor infertility and **IUA**.

Nordberg et al. conducted a biomechanical evaluation of tissue-engineered neo-uteri constructed using PGA scaffolds coated with poly-DL-lactide-co-glycolide and seeded with autologous endometrial and myometrial cells. These constructs were orthotopically implanted into rabbits and demonstrated the ability to support live births, thereby establishing their functional relevance. Over 6 months post-implantation, these engineered uteri progressively acquired mechanical properties closely resembling those of native uterine tissue as substantiated by tensile relaxation and strain-to-failure analyses 96. The seeded constructs exhibited significantly increased stiffness, elasticity, and viscoelastic behavior compared to non-seeded controls over time. This maturation of the engineered uteri was attributed to the establishment of a well-organized uterine-like architecture and ECM remodeling, highlighting the essential role played by PGA-based scaffolds in structural support and regenerative guidance. These findings validate PGA as a mechanically competent and biologically favorable platform for reconstructing engineered uteri and pave the way for future uterine factor infertility treatments.

Amiri et al. assessed the potential of PGA as an animal-free, synthetic scaffold for the 3D culture of human endometrial cells with the aim of mimicking native tissue structure *in vitro*. Electrospun PGA supported the co-culture of human endometrial stromal and epithelial cells without inducing cytotoxicity. Additionally, its porosity, fiber diameters, and biocompatibility were comparable to traditional fibrin-agarose scaffolds 97. Immunohistochemical analysis verified that stromal cells integrated well within the scaffold matrix and epithelial cells formed a surface monolayer closely resembling the *in vivo* structure of endometrial tissue. Although fibrin-agarose scaffolds showed slightly better epithelial clustering, PGA scaffolds facilitated robust cell proliferation and tissue-like organization. Given the environmental, ethical, and cost-related advantages of synthetic biomaterials over natural ones, this study identified PGA as a promising material for modeling endometrial tissues and regenerative applications.

MacKintosh et al. developed a 3D endometrial tissue model utilizing electrospun PGA scaffolds to mimic the architecture and function of bovine endometrial tissue. The PGA scaffold, with its porous, fibrous environment, facilitated the co-culture of primary endometrial stromal and epithelial cells and promoted the formation of a stratified, tissue-like construct 98. This model demonstrated histologically accurate cell layering, fibronectin deposition, and epithelial cell polarization, and cells responded functionally to physiological (oxytocin and arachidonic acid) and pathological (LPS) stimuli, as evidenced by the production of prostaglandins E2 and F2α. These results underscore the potential of PGA as a synthetic biomaterial for endometrial tissue modeling that provides a robust framework for investigating uterine physiology and disease mechanisms *in vitro*.

While PGA-based scaffolds have demonstrated remarkable promise in both *in vivo* uterine reconstruction and *in vitro* endometrial modeling, several critical considerations temper these encouraging results. For example, while Nordberg et al. reported live births in rabbits following implantation of PGA-based engineered uteri, Amiri et al. observed that fibrin-agarose scaffolds supported slightly superior epithelial clustering compared to PGA, suggesting that without additional biochemical cues, PGA may be less effective in promoting epithelial organization. Similarly, while PGA constructs reliably supported stromal-epithelial co-culture and stratification, outcomes such as ECM remodeling and mechanical maturation varied, depending on scaffold coatings, seeding strategies, and cell sources, highlighting methodological heterogeneity across studies. Moreover, although animal models have shown functional recovery, these settings may not fully recapitulate the cyclic hormonal dynamics, immune complexity, and vascularization demands of the human uterus, raising concerns about translational predictability. PGA's relatively rapid degradation profile also presents a double-edged sword: while advantageous for avoiding chronic foreign body responses, unless modified or combined with more durable polymers, it may compromise long-term mechanical stability. In addition, large-scale, GMP-compliant fabrication of electrospun PGA scaffolds with consistent fiber alignment, porosity, and mechanical properties remains an unresolved barrier. Collectively, these findings underscore that while PGA offers a structurally robust and ethically advantageous synthetic alternative to natural scaffolds, future efforts must focus on comparative head-to-head studies, standardized manufacturing protocols, long-term functional assessments in large-animal models, and rigorous regulatory evaluation to clarify its clinical viability for endometrial regeneration and infertility treatment.

Despite several reports demonstrating the application of PGA scaffolds to endometrial reconstruction, there is currently no direct evidence elucidating how PGA itself modulates intracellular signaling pathways or gene expression in uterine cells. Thus, while PGA provides mechanical support and serves as a promising biomaterial for structural repair, to fully establish its therapeutic potential in endometrial regeneration, future studies are required to clarify its bioactive roles at the molecular and cellular levels.

#### 2.2.3. PLA and endometrial regeneration

Polylactic acid (PLA), an FDA-approved biocompatible aliphatic polyester, has emerged as a promising synthetic biomaterial for endometrial tissue engineering due to its biodegradability, mechanical strength, and tunable degradation kinetics 99, 100. Despite its inherent hydrophobicity, PLA can be structurally modified or incorporated into composite systems to enhance its bioactivity and therapeutic functionality 101. PLA-based systems have been shown to support epithelial repair, modulate fibrotic responses, and promote the sustained release of growth factors 102, 103, which collectively enhance endometrial receptivity and functional recovery. In this context, Leprince et al. developed a novel degradable intrauterine medical device aimed at preventing the formation and recurrence of IUAs. This device uses a triblock copolymer consisting of PLA and high molecular weight PEO (polyethylene oxide), and overcomes the shortcomings of existing anti-adhesion barriers due to the fine-tuning of degradation time, swelling properties, and clinical usability for gynecological procedures. In a rat IUA model, the PLA-PEO-PLA prototype significantly achieved complete uterine re-epithelialization within the critical 5-6-day window, thus preventing the formation of fibrotic tissue and restoring uterine morphology 104. The carefully tailored degradation profile and physical flexibility of this material facilitated minimally invasive insertion and effective uterine wall coverage, thus demonstrating potential for future clinical applications in reproductive medicine. These results underscore the value of PLA-based copolymers as intelligent, bioresponsive platforms for endometrial regeneration and adhesion prevention.

Abraham et al. developed a controlled-release platform that enhances endometrial angiogenesis by encapsulating VEGF121 within PLA microparticles. Recognizing the limitations of short half-life and potential side effects of VEGF at high doses, the team designed a PLA carrier system for sustained release that delivered VEGF in a controlled manner to human endometrial stromal cells (HESCs). *In vitro* assays revealed that free and PLA-encapsulated VEGF121 markedly enhanced cell proliferation and migration and that PLA-mediated delivery maintained activity for 30 days 105. At the molecular level, the treatment-initiated activation of the PI3K/AKT pathway elevated the expressions of α-SMA and VEGFR2 and encouraged angiogenic remodeling. These results suggest a potential role for the VEGF121-PLA system in smooth muscle differentiation and vascular regeneration. These findings highlight the effectiveness of PLA-based VEGF delivery as a pro-angiogenic strategy for the repair of thin endometrium and demonstrate that this system offers a biocompatible and effective means of improving endometrial receptivity and fertility outcomes.

Although PLA-based scaffolds and delivery systems demonstrate promising outcomes in preventing IUA and promoting angiogenesis, several critical limitations and inconsistencies merit attention. For example, while Leprince et al. showed that a PLA-PEO-PLA copolymer device enabled rapid re-epithelialization and effective adhesion prevention, its efficacy was evaluated only in short-term rodent models; whether such results can be replicated in large-animal or hormonally dynamic settings remains uncertain. Conversely, while Abraham et al.'s VEGF-loaded PLA microparticles provided sustained pro-angiogenic activity, the balance between therapeutic efficacy and risks of aberrant vascularization or fibrosis is not well established, raising concerns about long-term safety. Furthermore, if not carefully engineered, the hydrophobicity of PLA—although partially addressed through copolymerization or composite design—may still hinder uniform cell adhesion and tissue integration. Methodologically, heterogeneity in scaffold formulations, degradation kinetics, and injury models complicates direct comparison across studies, and obscures consensus regarding optimal PLA designs. Translationally, challenges remain with respect to scaling GMP-compliant production, ensuring reproducibility of degradation rates, and gaining regulatory approval for devices that blur the boundaries between drug-delivery systems and implantable biomaterials. Collectively, while PLA-based platforms highlight the potential of intelligent, bioresponsive scaffolds for endometrial regeneration, future work must focus on standardized comparative studies, long-term functional validation, and rigorous clinical translation strategies to clarify their role in reproductive medicine.

Although PLA scaffolds have been investigated for endometrial reconstruction, to date, no direct evidence has clarified how PLA intrinsically modulates signaling pathways or gene expression in uterine cells. At present, PLA is primarily recognized for its structural and drug delivery capacities, while its bioactive influence on cellular behavior remains largely unexplored. Future mechanistic studies will therefore be essential to determine whether PLA functions solely as a passive scaffold, or can also act as a bioinstructive material in endometrial regeneration.

#### 2.2.4. Poly (lactic-co-glycolic acid) and endometrial regeneration

Poly (lactic-co-glycolic acid) (PLGA) is a well-characterized, FDA-approved synthetic copolymer widely recognized for its outstanding biocompatibility, tunable degradation rates, and capacity for sustained drug delivery 106. These attributes render it a versatile scaffold and carrier system in the field of endometrial tissue engineering, particularly for addressing pathological conditions such as thin endometrium and IUA. The proficiency of PLGA at encapsulating a diverse array of bioactive agents, including small molecules, hormones, and stem cells, facilitates targeted, controlled drug delivery within tissue microenvironments 107, 108. Furthermore, recent innovations that exploit its responsive behavior and multifunctional potential have extended its applications in personalized and minimally invasive treatments for endometrial dysfunction and uterine factor infertility.

Raheem et al. explored the therapeutic efficacy of PLGA nanoparticles loaded with pentoxifylline (PTXF) for treating thin endometrium in a rat model. Given the anti-inflammatory and angiogenic properties of PTXF, they focused on enhancing its delivery and bioavailability by encapsulating it in PLGA. In this study, rats with ethanol-induced endometrial thinning were treated with free PTXF, PLGA alone, or PLGA-PTXF nanoparticles 109. Histopathological analyses demonstrated that PLGA-PTXF significantly outperformed the other treatments by improving endometrial and myometrial thickness, promoting vascular and glandular regeneration, and reducing inflammation. These enhanced outcomes were attributed to the controlled release, targeted delivery, and antioxidative benefits conferred by PLGA-PTXF. These findings emphasize the potential of PLGA-based nanoparticle systems as robust platforms for endometrial regeneration therapies, particularly for addressing uterine factor infertility and IUA.

Chen et al. engineered a biomimetic, multi-scale scaffold for uterine tissue regeneration produced by integrating high-resolution 3D printing with stimuli-responsive polymer systems and sustained-release drug delivery platforms. To mimic the biomechanical properties of myometrium, they used a PLATMC-PLGA polymer composite. In addition, layered coatings composed of polydopamine, estradiol (E2), and HA were applied to enable the precise, controlled release of the bioactive agents 110. To further boost regenerative efficacy, the scaffolds were enhanced with hydrogels encapsulating bone marrow-derived mesenchymal stem cells to promote cell viability and functional tissue reconstruction. Notably, the constructs exhibited thermally responsive shape-shifting behavior, transitioning from planar forms to tubular configurations, underscoring the potential of 3D printing technologies to produce dynamic, physiologically relevant architectures for uterine tissue engineering.

Prakapenka et al. developed a hormone delivery system using PLGA nanoparticles encapsulating 17β-estradiol (E2) to enhance bioavailability and therapeutic efficacy for the treatment of menopausal symptoms. Notably, this approach resulted in increased uterine stimulation, a peripheral effect associated with elevated estrogen exposure. The efficacy of E2-loaded PLGA nanoparticles was demonstrated in ovariectomized rats, which showed improved spatial memory and significantly greater uterine stimulation than when free E2 was administered 111. Furthermore, the sustained-release characteristics of PLGA prolonged systemic E2 exposure, thereby enhancing its physiological effects on cognition and reproductive tissues. These findings illustrate the dual effect of PLGA-based carriers, viz., and highlight the need for caution due to elevated peripheral exposure risks.

**Figure 3:**
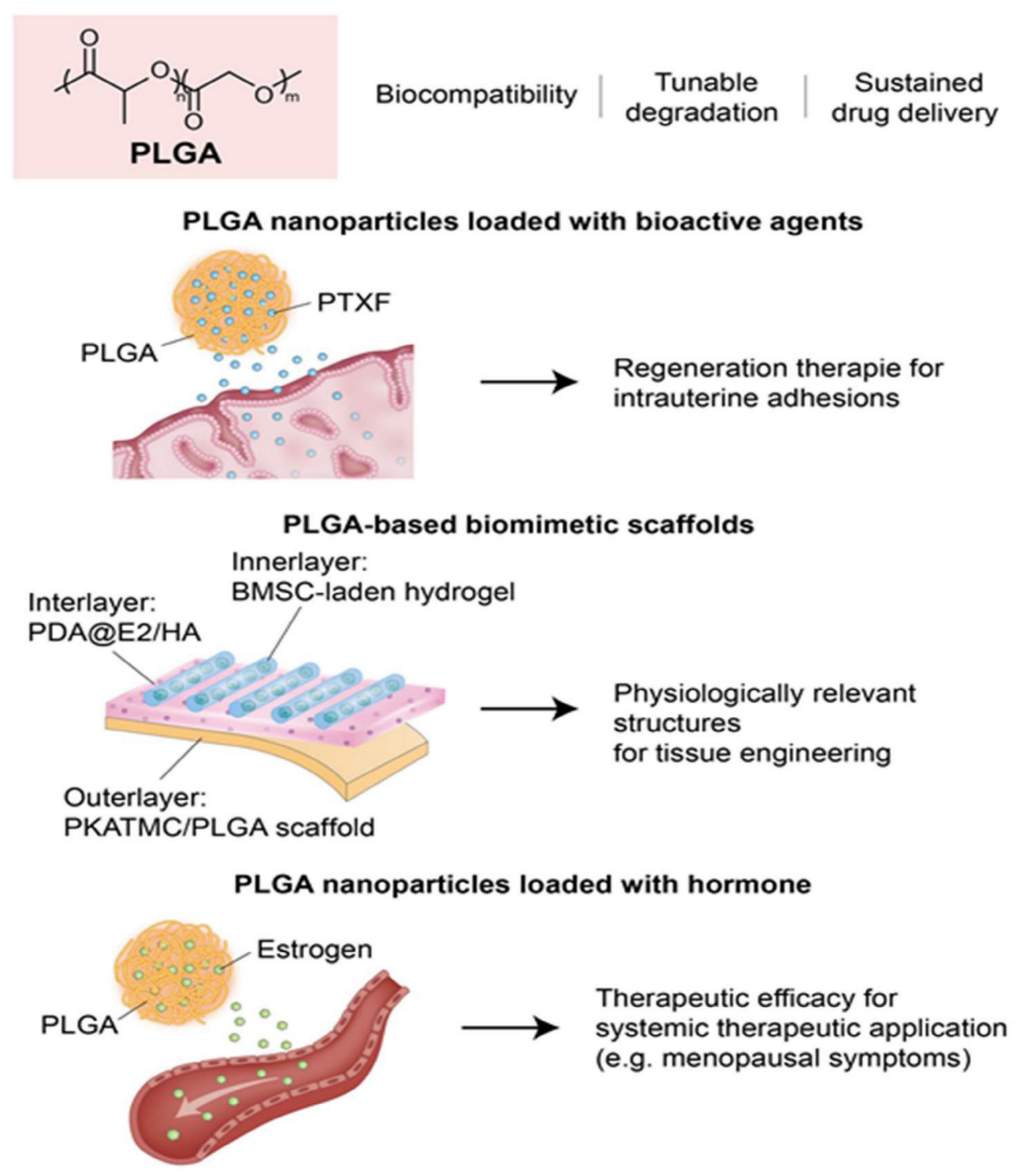
** Poly (lactic-co-glycolic acid) (PLGA)-based platforms for endometrial regeneration**. PLGA offers a versatile foundation for both sustained drug delivery and structural support in uterine tissue engineering. (Left) PLGA nanoparticles loaded with bioactive agents such as pentoxifylline or estradiol enable controlled, localized release, promoting endometrial thickening, angiogenesis, and reduced inflammation. (Center) PLGA-based composites combined with 3D printing and mesenchymal stem cell-laden hydrogels form biomimetic scaffolds that adapt to uterine architecture and enhance functional regeneration. (Right) Hormone-loaded PLGA systems support systemic therapeutic applications, with potential benefits for reproductive tissues and neuroendocrine regulation. Together, these strategies highlight the multifaceted role of PLGA in restoring endometrial structure and function, offering translational potential in the treatment of uterine factor infertility.

These studies collectively underscore the potential of PLGA-based systems in endometrial regeneration due to their ability to act as structural scaffolds and intelligent drug delivery platforms. By enabling sustained and localized release of pro-regenerative agents, PLGA promotes epithelial repair, angiogenesis, and anti-inflammatory responses in damaged uterine tissue (Figure 3). Despite these promising advances, the application of PLGA in endometrial regeneration faces several unresolved challenges and conflicting outcomes. For example, while Raheem et al. demonstrated significant improvements in epithelial and stromal recovery using PLGA-PTXF nanoparticles, Prakapenka et al. reported that E2-loaded PLGA carriers led to unintended peripheral estrogen stimulation, raising concerns about off-target effects and long-term safety. Such findings underscore that the therapeutic benefit of PLGA often depends less on the polymer itself, and more on the encapsulated agent, complicating efforts to isolate the intrinsic contribution of PLGA to tissue regeneration. Moreover, heterogeneity in scaffold design—including nanoparticle formulations, copolymer ratios, and drug-loading efficiencies—introduces substantial variability, making direct cross-study comparisons difficult. Methodologically, most studies remain limited to small-animal models with short follow-up periods, which fail to capture the cyclical hormonal, immunological, and vascular dynamics of the human uterus. Translational barriers are also notable: although PLGA is FDA-approved and widely used in drug delivery, scaling GMP-compliant, reproducible constructs for uterine-specific applications remains a major challenge, particularly when combined with bioactive molecules or stem cells. Furthermore, regulatory approval pathways for multifunctional PLGA-based systems—straddling the categories of medical devices and drug-delivery platforms—remain ambiguous. Collectively, while PLGA is an exceptionally versatile material, with strong potential as both scaffold and delivery vehicle, future efforts must emphasize standardized formulation, large-animal validation, long-term biosafety assessment, and harmonized regulatory strategies to clarify its true translational role in endometrial regeneration.

Although poly(lactic-co-glycolic acid) (PLGA) scaffolds have been widely explored for endometrial repair and drug delivery, there is a paucity of evidence that directly addresses how PLGA itself modulates signaling cascades or gene expression in uterine cells. To date, its regenerative benefits are largely attributed to its favorable biodegradability and capacity for the controlled release of bioactive agents, rather than any intrinsic bioactivity. Future mechanistic investigations are therefore required to elucidate whether PLGA merely serves as a passive carrier, or also exerts direct regulatory effects on molecular pathways governing inflammation, fibrosis, and angiogenesis in endometrial regeneration.

#### 2.2.5. Critical summary and outlook for synthetic biomaterials-based endometrial regeneration

Synthetic biomaterials, such as PCL, PGA, PLA, and PLGA, offer tunable mechanical properties and controlled degradation profiles, making them attractive candidates for endometrial regeneration. Compared with natural polymers, synthetic scaffolds provide superior structural stability, scalability, and reproducibility, which are essential for clinical translation. However, their intrinsic bioactivity remains limited, and regenerative outcomes are often achieved only after surface modification, incorporation of bioactive ligands, or combination with stem cells and growth factors. Mechanistically, PCL and its composites have been shown to downregulate IL-6 while upregulating IL-10, VEGFA, VEGFB, and TGF-β, thereby suppressing inflammation and promoting angiogenesis and stromal remodeling. In contrast, while PGA, PLA, and PLGA have been successfully employed in uterine repair models, direct evidence of their ability to regulate signaling cascades or gene expression in uterine cells remains lacking. The regenerative benefits of PGA, PLA, and PLGA are largely attributed to their biodegradability, drug delivery capacity, and mechanical support, rather than any intrinsic cellular modulation.

When comparing synthetic and natural biomaterials, synthetic polymers excel in mechanical strength, reproducibility, and tunable degradation rates, whereas natural biomaterials more effectively engage with cell receptors and modulate signaling pathways. This distinction highlights the potential of hybrid or composite systems that integrate the structural advantages of synthetic scaffolds with the biochemical functionality of natural polymers. Moving forward, research should prioritize (i) elucidating the direct molecular interactions of synthetic scaffolds with uterine cells, (ii) standardizing head-to-head comparative studies across material classes, and (iii) developing smart composite platforms that combine structural fidelity, controlled release, and bioactive signaling capacity. Such strategies will be critical to overcome current translational barriers and achieve clinically viable solutions for endometrial regeneration.

## 2. Tissue engineering-based endometrial regeneration

The endometrium, which lines the uterus, is critical for reproductive health as it plays an essential role in both the menstrual cycle and pregnancy. It primarily supports the implantation of a fertilized egg, making its integrity crucial for successful conception and fetal development 112. Conditions such as IUA can impair endometrial function, leading to infertility and recurrent miscarriages 113. The remarkable ability of the endometrium to regenerate each month makes it an optimal target for regenerative medicine, as tissue engineering techniques provide promising methods for repairing or regenerating damaged endometrial tissue using scaffolds, cells, and bioactive molecules 114. Building upon the natural and synthetic biomaterials discussed in Section 1, which provide the essential structural and biochemical foundations for endometrial repair, Section 2 focuses on how these materials have been integrated into advanced tissue engineering strategies. Specifically, recent developments in scaffold-stem cell composites, bioactive hydrogels, and decellularized extracellular matrix systems illustrate how biomaterial platforms can be translated into functional regenerative technologies for the uterus. By leveraging the intrinsic properties of polymers, such as collagen, gelatin, alginate, hyaluronic acid, and synthetic polyesters, researchers have designed multifaceted constructs that restore structural integrity, while also recapitulating hormonal responsiveness, immunomodulation, and angiogenic signaling. This section highlights the latest progress in these biomaterial-driven approaches, emphasizing their translational potential to restore endometrial function and fertility.

### 2.1. Endometrial regeneration based on integrating biomaterials and various types of stem cells

Current advancements in regenerative medicine have shown that combining biomaterials with stem cell therapies provides an effective means of restoring the structural and functional integrity of the endometrium, especially for IUA or thin endometrium nonresponsive to traditional treatments. Biomaterial scaffolds, which range from natural hydrogels to decellularized ECM constructs, not only provide mechanical support but also create dynamic microenvironments that promote stem cell survival, localization, paracrine signaling, and immunomodulation. When integrated with various stem cell sources, such as adipose- or umbilical cord-derived or amniotic or endometrial stem cells, these platforms facilitate targeted delivery and provide sustained therapeutic impacts, leading to enhanced endometrial thickness, glandular restoration, neovascularization, and embryo implantation.

Zheng et al. developed an innovative strategy for endometrial regeneration by combining biomaterials with stem cell therapy, and demonstrated the therapeutic potential of a redox-responsive, injectable hydrogel composed of thiolated hyaluronan and chitosan (tHA-tChi). This antioxidant hydrogel was engineered to co-deliver adipose-derived stem cells (ADSCs) and platelet-rich plasma (PRP), thereby achieving sustained growth factor release and enhancing stem cell viability within the oxidative microenvironment of damaged endometrial tissue 115. *In vitro* and *in vivo* studies showed that the ADSCs/tHA-tChi/PRP composite significantly promoted angiogenesis, reduced fibrosis, and restored endometrial thickness and receptivity by activating the VEGF/AKT/BAD signaling pathway. Notably, synergy between this composite and stem cells led to a marked improvement in pregnancy outcomes in a mouse model of thin endometrium, demonstrating the potential of multifunctional biomaterials as delivery platforms that enhance the efficacy of stem cell-based regenerative therapies.

Li et al. developed an innovative injectable sodium alginate-based hydrogel platform optimized for stem cell-mediated endometrial repair. Employing calcium gluconate as the crosslinking agent, the team achieved enhanced injectability and uniform gelation compared to traditional calcium chloride systems. The hydrogel was further functionalized with RGD peptides to enhance cellular adhesion and promote the proliferation and differentiation of encapsulated umbilical cord-derived mesenchymal stem cells (UCMSCs). In a murine model of endometrial injury, intrauterine delivery of this RGD-modified hydrogel, containing UCMSCs, significantly improved endometrial regeneration, reduced fibrotic tissue formation, stimulated neovascularization, and markedly increased pregnancy rates compared to untreated and UCMSC-only groups 116. The study underscored the therapeutic potential of bioactive, stem cell-laden injectable hydrogels as a minimally invasive strategy for restoring uterine function and improving fertility outcomes.

Hu et al. developed a minimally invasive therapeutic strategy for IUA by incorporating human umbilical cord-derived mesenchymal stem cells (UCMSCs) into a bioorthogonal *in situ*-gelling HA-based hydrogel using the Diels-Alder click reaction. This injectable biomaterial conformed to the uterine architecture and provided a supportive microenvironment for UCMSCs, thus preserving cell viability and enhancing paracrine-mediated effects. The composite hydrogel not only facilitated cell proliferation, migration, angiogenesis, and antifibrotic activity *in vitro*, but also significantly restored endometrial morphology, receptivity, and fertility *in vivo* 77. In addition, the hydrogel promoted macrophage recruitment and M2 polarization, highlighting the immunomodulatory synergy between the UCMSC containing HA-based hydrogel and stem cells. These findings emphasize the translational promise offered by biomaterial-assisted stem cell delivery systems for uterine regeneration and reproductive restoration.

Zhang et al. developed a regenerative strategy for endometrial injury by integrating hUCMSCs with an injectable, self-healing hydrogel composed of HA-CHO and hydrazide-grafted gelatin (Gel-ADH). This HA/Gel hydrogel provided a supportive 3D matrix that enhanced hUCMSC viability, retention, and paracrine activity within the uterine cavity 65. In a rat model of endometrial injury, this hydrogel-hUCMSC composite significantly promoted tissue regeneration by reducing fibrosis, stimulating angiogenesis, and restoring glandular architecture and endometrial receptivity. Furthermore, treatment activated the MEK/ERK1/2 signaling pathway and balanced pro- and anti-inflammatory cytokine expression, and thus, improved embryo implantation and successful live births. These findings emphasize the therapeutic synergistic effect of biomaterial-stem cell integration in restoring endometrial function and fertility.

Huang et al. demonstrated the regenerative efficacy of integrating human amniotic mesenchymal stem cells (hAMSCs) and a thermoresponsive hydrogel scaffold composed of poly(polyethylene glycol citrate-co-N-isopropylacrylamide) mixed with gelatin (PPCNg) for the treatment of IUA. This PPCNg hydrogel provided a biocompatible, minimally invasive delivery platform that preserved the stemness, viability, and multilineage differentiation potential of hAMSCs, and enhanced their retention and colonization within the uterine cavity 117. *In vivo* transplantation of PPCNg/hAMSCs in a rat model of endometrial injury significantly improved endometrial morphology, reduced fibrosis, stimulated angiogenesis, and upregulated receptivity markers such as ER and PR. Furthermore, this biomaterial-stem cell synergy led to a significant restoration of fertility by increasing pregnancy rates and embryo implantation. The study highlights the critical role that biomaterial-assisted stem cell delivery systems can play in advancing functional uterine regeneration and improving reproductive outcomes.

Park et al. engineered a bioinspired 3D artificial endometrium by integrating endometrial stem cells with a multilayered scaffold composed of natural biodegradable biomaterials (collagen, HA, and agarose) to replicate the structural and functional complexity of native endometrial tissue. The construct incorporated vascular endothelial cells, stromal fibroblasts, and endometrial stem cells in distinct layers mimicking the mucosal, stromal, and basal zones of the uterus, respectively 118. This biomimetic platform demonstrated excellent biocompatibility, mechanical integrity, and hormone responsiveness, while maintaining cell viability, metabolic activity, and endometrial-specific marker expression. Notably, transplantation of the stem cell-laden artificial endometrium into a murine model of uterine injury led to endometrial regeneration, fertility restoration, and live births without chromosomal abnormalities. This study underscores the crucial role of biomaterial-stem cell synergy in replicating the endometrial microenvironment and advancing therapeutic strategies for reproductive medicine.

**Figure 4:**
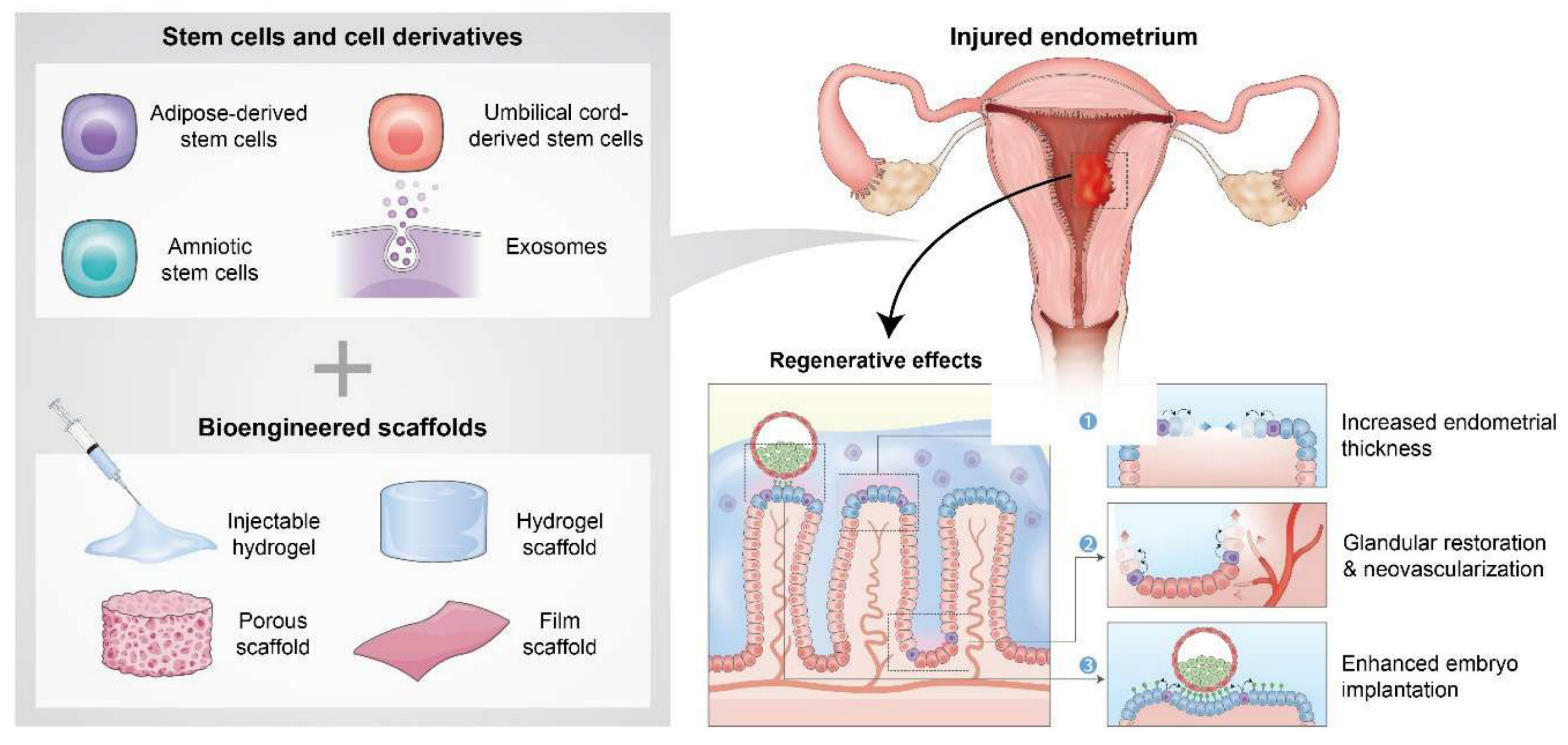
** Strategies integrating biomaterials and stem cells for endometrial regeneration.** This schematic summarizes advanced approaches combining various stem cell types with bioengineered scaffolds for restoring damaged endometrial tissue. Injectable hydrogels composed of hyaluronic acid, gelatin, alginate, or composite matrices serve as delivery vehicles for stem cells—including adipose-derived (ADSCs), umbilical cord-derived (UCMSCs), amniotic (hAMSCs), and endometrial stem cells—enhancing their survival, retention, and paracrine activity. These cell-laden constructs promote epithelial regeneration, angiogenesis, and anti-fibrotic effects via pathways, and modulate immune responses through M2 macrophage polarization. Additionally, exosome-loaded scaffolds offer cell-free alternatives with comparable regenerative efficacy. Collectively, these integrated platforms improve endometrial receptivity, embryo implantation, and fertility outcomes, underscoring their translational promise in reproductive regenerative medicine.

Xin et al. developed a cell-free regenerative strategy by incorporating mesenchymal stem cell (MSC)-derived exosomes into a porous collagen scaffold (CS/Exos) to promote endometrial repair and fertility restoration through immunomodulation. This bioengineered construct enabled sustained exosome release and enhanced exosome retention at sites of injury, overcoming the limitations of free exosome delivery. In a rat model of endometrial damage, CS/Exos significantly improved endometrial thickness, glandular regeneration, epithelial proliferation, neovascularization, and hormonal receptor expression, ultimately restoring successful embryo implantation and pregnancies 119. Mechanistically, the exosome-loaded scaffold enhanced the recruitment and polarization of macrophages toward an M2 anti-inflammatory phenotype via miRNA-mediated signaling (particularly through miR-223-3p), which reduced inflammation and created a favorable microenvironment for tissue repair. These findings highlight the synergistic therapeutic potential of biomaterials and stem cell-derived bioactive agents in promoting clinically viable interventions for IUA and reproductive disorders. Collectively, the integration of stem cells and bioengineered scaffolds has emerged as an effective strategy for enhancing endometrial regeneration and restoring fertility. These approaches synergistically modulate local microenvironments, reduce fibrosis, and promote tissue remodeling (Figure 4). Continued progress in scaffold design and cell selection is crucial to optimize clinical translation. Future efforts should focus on developing scalable, immunocompatible platforms tailored for individual reproductive repair.

While biomaterial-stem cell integration strategies consistently demonstrate synergistic effects in simultaneously promoting angiogenesis, reducing fibrosis, and restoring fertility, several critical issues remain unresolved. First, outcomes vary depending on stem cell sources: for example, UCMSCs and hAMSCs have shown strong paracrine and immunomodulatory effects, whereas endometrium-derived stem cells may offer superior tissue specificity, but present challenges in availability and expansion. Similarly, although exosome-loaded scaffolds provide a cell-free alternative with promising immunomodulatory benefits, their therapeutic durability and scalability remain uncertain, compared to live stem cell delivery. Second, methodological limitations complicate interpretation across studies. Many reports rely on rodent IUA or chemically induced thin endometrium models that fail to reproduce the complex hormonal cycling, vascular dynamics, and immune heterogeneity of the human uterus, limiting translational predictability. Also, variations in hydrogel crosslinking chemistry, mechanical strength, and degradation kinetics make it difficult to establish consensus regarding optimal scaffold design. Third, conflicting findings highlight unresolved questions: while some studies report robust fertility restoration and live births following scaffold-stem cell transplantation, others demonstrate only partial recovery or fail to achieve functional outcomes, raising concerns about reproducibility. Finally, clinical translation is constrained by manufacturing, regulatory, and safety barriers. The GMP-compliant large-scale production of stem cell-laden biomaterials, long-term safety data addressing risks such as ectopic differentiation or tumorigenesis, and ambiguous approval pathways for combination products all remain major challenges. Taken together, these findings emphasize that while biomaterial-stem cell synergy represents a transformative strategy for endometrial regeneration, future research must prioritize comparative studies across stem cell sources, standardized scaffold design, large-animal and long-term safety validation, and harmonized regulatory frameworks, to advance these approaches toward reliable clinical application.

### 2.2. Endometrial regeneration by cell-free scaffolds or hydrogels

Huang et al. developed a multilayered gelatin/polycaprolactone (GT/PCL) gradient biofilm scaffold to repair extensive endometrial and myometrial injuries in a rabbit model, thereby overcoming the limitations associated with small-animal studies. This surgically sutured scaffold facilitated anatomically guided tissue regeneration, leading to complete restoration of the uterine cavity within 28 days. Histological analyses confirmed the reestablishment of a functional endometrium with glandular and vascular structures, along with organized smooth muscle fibers that composed the native circular and longitudinal architecture of the uterus 120. The repaired tissue successfully supported embryo implantation and subsequent placental development. Furthermore, comprehensive four-dimensional (4D), label-free proteomic profiling revealed the activation of multiple key pathways, including the cell adhesion, ferroptosis, phagosome activity, Rap1 and HIF-1 signaling, and energy metabolism pathways, indicating a coordinated molecular response that drives endogenous tissue repair. These findings highlight the potential of biomaterial-guided regeneration strategies in large-scale uterine reconstruction and functional fertility restoration.

Yoshimasa et al. developed a decellularized ECM scaffold (DES) derived from rat endometrium that promoted comprehensive endometrial regeneration following extensive stromal ablation. By integrating this biomaterial scaffold with a silicone tube to prevent intrauterine adhesion, the authors demonstrated that DES significantly enhanced stromal regrowth compared to controls, as evidenced by increased stromal area and cell density 121. In addition, DES preserved essential structural matrix proteins, such as collagen I and laminin, while removing cellular components, producing a microenvironment conducive to host cell infiltration. Although epithelial regeneration was incomplete, likely due to physical interference by the silicone tube and a lack of epithelial progenitors or bioactive cues, the results highlighted the potential of poECM-based scaffolds for reconstructing structurally compromised endometrial tissue. This study establishes a foundational platform for future strategies that incorporate biomaterials with epithelial stem cells or molecular factors to achieve comprehensive, multilayered endometrial restoration.

Xin et al. introduced a cell-free regenerative platform by engineering an acellular, bioactive scaffold composed of stromal-derived factor-1 alpha (SDF-1α)-loaded nanoparticles embedded in E7-modified collagen (CES) to promote endometrial repair and fertility restoration. This multifunctional biomaterial synergistically enhanced the recruitment of endogenous mesenchymal stem cells (MSCs) via macrophage-mediated signaling and steered their immunomodulatory activities toward tissue regeneration 122. In acute and fibrotic rat models of intrauterine adhesion, CES treatment significantly enhanced endometrial thickness, gland formation, hormonal responsiveness, and pregnancy outcomes, surpassing traditional stem cell transplantation approaches. The study underscored the translational potential of the biomaterial-directed activation of endogenous stem cells as a clinically viable alternative to exogenous cell therapy in uterine regenerative medicine.

Chen et al. developed a bioactive, cell-free scaffold by combining decellularized human amniotic ECM (HAECM) with 17β-estradiol (E2)-loaded PLGA microspheres to achieve sustained intrauterine hormone delivery and facilitate endometrial regeneration. This composite scaffold retained a highly porous architecture, promoted endometrial cell proliferation, and enabled controlled E2 release over 21 days, thereby mimicking the female menstrual cycle 123. The HAECM scaffold itself supported biologic functionality by increasing the expressions of EGF and IGF-1, while the integration of E2 microspheres synergistically enhanced cytokine signaling and cellular proliferation. Notably, the hybrid system minimized burst release and provided mechanical support and biochemical cues to restore endometrial function. These findings highlight the therapeutic potential of combining natural ECM-derived scaffolds with hormone-loaded delivery systems to create a pro-regenerative microenvironment for uterine tissue repair.

Yao et al. developed a multifunctional, thermosensitive hydrogel system by embedding β-estradiol-loaded, decellularized uterus-derived ECM nanoparticles (E2@uECMNPs) into an aloe vera-poloxamer scaffold (AP hydrogel) to promote endometrial regeneration and prevent intrauterine adhesion. This bioresponsive platform facilitated localized, sustained hormone delivery while simultaneously exploiting the pro-regenerative and immunomodulatory properties of natural ECM 124. In a rat model of endometrial injury, intrauterine administration of the E2@uECMNPs/AP hydrogel significantly enhanced epithelial regeneration, reduced fibrosis, upregulated the expressions of Ki67 and ER-β, and suppressed pro-inflammatory cytokines such as TNF-α and TGF-β1.

Interestingly, synergism between the bioactive matrix-derived nanoparticles and the thermoresponsive scaffold supported uterine morphological and functional recovery more effectively than estradiol alone or commercial formulations. This study highlights the therapeutic potential of integrating endogenous ECM-based biomaterials with controlled drug delivery systems for advanced uterine tissue engineering.

Zhang et al. demonstrated that the urinary bladder matrix (UBM), a decellularized ECM derived from porcine tissue, serves as an effective biomaterial scaffold for enhancing endometrial regeneration in a rat model of IUA. This scaffold promoted substantial restoration of endometrial architecture, which included increasing thickness and promoting gland formation, angiogenesis, and epithelial proliferation, while concurrently reducing fibrosis and proinflammatory cytokine expression 125. Furthermore, UBM implantation upregulated key receptivity markers, including integrin αVβ3 and leukemia inhibitory factor (LIF), and thus, improved embryo implantation rates and pregnancy outcomes. These findings emphasize the potential of bioactive ECM scaffolds to initiate endogenous repair mechanisms and restore uterine function, thus supporting their application in fertility-preserving therapies that do not rely on exogenous stem cell transplantation.

Although cell-free scaffolds and hydrogels have demonstrated considerable potential by harnessing endogenous repair mechanisms, several limitations and conflicting outcomes warrant critical appraisal. For example, while Xin et al. reported that SDF-1α-loaded scaffolds outperformed stem cell transplantation in recruiting host MSCs and improving fertility, Yoshimasa et al. observed incomplete epithelial regeneration with decellularized scaffolds, suggesting that ECM-based constructs alone may lack sufficient cues for full functional recovery. Similarly, while hormone-loaded hydrogel systems (e.g., Chen et al., Yao et al.) provide controlled release and enhance endometrial repair, questions remain regarding long-term safety, potential systemic hormone exposure, and whether such benefits can be sustained in hormonally dynamic human uterine environments. Methodological variability across studies—including differences in scaffold source (e.g., porcine UBM *vs.* human amniotic ECM), crosslinking chemistries, and animal models—complicates cross-comparison and weakens consensus on optimal design parameters. Importantly, most findings are derived from rodent models with relatively short observation windows, which may fail to capture cyclical hormonal regulation, pregnancy-related remodeling, or long-term fibrotic recurrence. Translational challenges also remain substantial: donor-to-donor variability in ECM scaffolds, batch-to-batch inconsistency in decellularization, and regulatory ambiguity regarding xenogeneic materials all impede clinical scalability. Taken together, while cell-free biomaterial systems circumvent the challenges of stem cell transplantation and offer a promising platform for minimally invasive uterine repair, to fully realize their clinical potential, future work must focus on standardized scaffold processing, large-animal validation, functional fertility endpoints, and harmonized regulatory frameworks.

### 2.3. Tissue engineering and endometriosis treatment

Endometriosis is a chronic, estrogen-dependent inflammatory disease characterized by the ectopic implantation and proliferation of endometrial tissue, causing pelvic pain and infertility. Conventional therapeutic approaches, including hormonal therapies and surgical resection, have limited efficacy, cause side effects, and show high recurrence rates. While most of the preceding subsections have focused on strategies to restore endometrial integrity in conditions such as IUA or thin endometrium, endometriosis represents a unique, but equally relevant, clinical context for tissue engineering. Unlike fibrotic or atrophic disorders that are characterized by insufficient regeneration, endometriosis involves aberrant proliferation, ectopic implantation, and pathological remodeling of endometrial tissue. This pathological “over-regeneration” shares fundamental mechanisms with physiological repair, such as ECM remodeling, angiogenesis, and immune modulation, but occurs in a dysregulated microenvironment. Therefore, incorporating tissue engineering approaches into endometriosis research expands the translational scope of endometrial bioengineering, while also providing a valuable framework to dissect the molecular and cellular processes that distinguish normal regeneration from pathological remodeling. This perspective ensures that the discussion of endometriosis remains consistent with the overarching theme of this review—leveraging biomaterials and engineering platforms to modulate endometrial regeneration and improve reproductive health.

**Figure 5:**
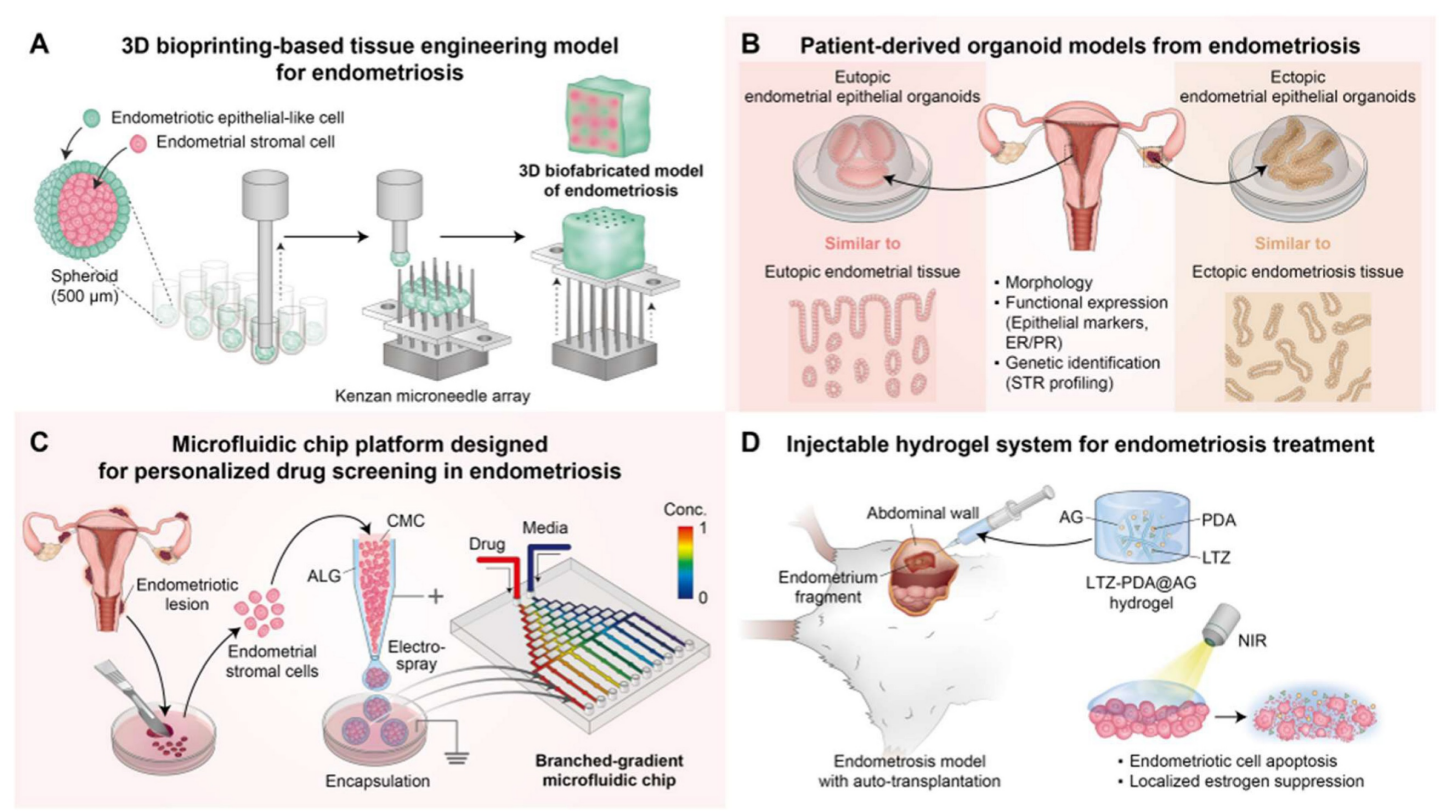
Tissue engineering-based platforms for modeling and treating endometriosis. *Scaffold-free 3D bioprinted model of endometriosis* using the Kenzan method. Endometriotic spheroids composed of 12Z epithelial-like cells and T-HESC stromal cells were assembled into complex multicellular constructs without exogenous scaffolds. The model mimics the inflammatory and hormonal microenvironment of endometriosis, enabling analysis of cell-cell interactions and drug response (A). *Patient-derived organoid models* from eutopic and ectopic endometrial tissues faithfully replicate morphological, molecular, and hormonal features of ovarian endometriosis. EUT-O and ECT-O organoids exhibit distinct growth patterns, hormone receptor expression, and secretory activity, establishing a physiologically relevant platform for personalized therapeutic testing (B). *Microfluidic chip-based drug screening platform* integrates hESC-laden hydrogel microcapsules within a branched-gradient system to enable patient-specific, high-throughput drug testing. The platform captures individual variability in therapeutic response and enables evaluation of both conventional hormonal agents and novel compounds (C). *Injectable, photothermal-responsive hydrogel system (LTZ-PDA@AG)* combines polydopamine nanoparticles and letrozole within an agarose matrix for NIR-controlled drug release. Upon NIR irradiation, the hydrogel triggers localized hyperthermia and estrogen suppression, synergistically inducing apoptosis in endometriotic stromal cells while offering biocompatibility and controlled biodegradation (D).

Wendel et al. developed a novel 3D bioprinting-based tissue engineering model for endometriosis using the scaffold-free Kenzan method to biofabricate complex, multicellular constructs that mimic the endometriotic microenvironment 126. This study established the first scaffold-free, biofabricated 3D model of endometriosis using bioprinting technologies, providing a physiologically relevant platform to study epithelial-stromal interactions, hormonal and inflammatory responses, and advance therapeutic screening. The researchers optimized spheroid production using the 12Z endometriotic epithelial-like cell line and T-HESC endometrial stromal cells to achieve spheroids (~500 µm) that maintained endometriosis-relevant characteristics, including elevated inflammatory cytokine secretion and estrogen-related gene expression 126. These spheroids were subsequently assembled into larger, scaffold-free 3D constructs via the Kenzan microneedle array, which enabled controlled spatial organization without exogenous biomaterials (Figure 5A).

Zhang et al. developed patient-derived organoid models from eutopic and ectopic endometrial tissues to replicate ovarian endometriosis, offering a novel tissue engineering platform for studying its pathogenesis and therapeutic response 127. Using tissues collected from ovarian endometriosis patients, they successfully established eutopic endometrial epithelial organoids (EUT-O) and ectopic endometrial epithelial organoids (ECT-O) within 3D Matrigel matrices. These organoids preserved the histological, molecular, and genetic features of their parental tissues, including the expression of epithelial markers (EPCAM, CK7, E-cadherin), estrogen and progesterone receptors (ER, PR), and secretory activity as evidenced by PAS staining. Genetic analysis confirmed 100% identity with the original tissues 127. Notably, the organoids exhibited distinct morphological and proliferative characteristics dependent on their eutopic or ectopic origins. EUT-O formed cystic structures and proliferated rapidly, while ECT-O displayed mixed or solid morphologies, slower growth, and lower establishment success rates. This study establishes endometriosis-derived epithelial organoids as physiologically relevant, patient-specific 3D models that closely mimic *in vivo* tissue architecture and hormone responsiveness (Figure 5B).

Chen et al. developed a novel tissue engineering-based microfluidic chip platform designed for personalized drug screening in endometriosis by leveraging patient-derived primary human ectopic endometrial stromal cells 128. The platform integrates hESC-laden hydrogel microcapsules, composed of a carboxymethyl cellulose (CMC) core and an alginate shell, into a branched-gradient microfluidic chip. This configuration replicates a dynamic 3D microenvironment, supports spheroid formation, and enables high-throughput, gradient-controlled drug testing. By exposing patient-specific microcapsules to different therapeutic agents, the system revealed significant inter-individual variability in drug response closely correlated with clinical outcomes and disease recurrence 128. Beyond conventional hormonal therapies (dienogest and dydrogesterone), novel compounds such as cilengitide and wortmannin were evaluated using the chip, revealing targeted anti-proliferative effects (Figure 5C).

Tian et al. developed an innovative injectable hydrogel system for endometriosis treatment by integrating tissue engineering with controlled drug delivery and photothermal therapy. The devised system, termed LTZ-PDA@AG hydrogel, consists of an agarose (AG) hydrogel matrix containing polydopamine (PDA) nanoparticles and the aromatase inhibitor letrozole (LTZ) 129. Designed for *in situ* application, the hydrogel allows precise, near-infrared (NIR)-controlled release of letrozole photothermal heating under NIR irradiation facilitated by the PDA. This photothermal effect directly induces endometriotic cell apoptosis and accelerates the localized release and diffusion of letrozole to achieve synergistic endocrine-photothermal therapy. *In vitro* studies using patient-derived endometrial stromal cells confirmed the ability of the hydrogel to promote apoptosis and suppress proliferation via estrogen suppression and photothermal effects 129. In addition, the hydrogel demonstrated excellent biocompatibility, biodegradability, and mechanical stability, and NIR-triggered heating accelerated its degradation, enabling controlled drug release and safe elimination (Figure 5D).

Anthis et al. developed a tissue engineering-based approach using stimuli-responsive, degradable hydrogel implants for reversible mechanical contraception and endometriosis treatment. Their innovative system targets the fallopian tubes, which play a central role in retrograde menstruation and the spread of endometrial cells into the peritoneal cavity—a key factor in the pathogenesis of endometriosis 130. Hydrogel implants, composed of superabsorbent acrylamide-based polymers crosslinked with photolabile or thiol-degradable linkers, were designed for the functional occlusion of fallopian tubes through swelling-mediated blockage, thereby preventing the passage of both sperm and endometrial cells 130. A proof-of-concept endometriosis prevention experiment demonstrated that hydrogel-blocked fallopian tubes effectively prevented the migration of lesion-forming endometrial cells under simulated retrograde menstruation conditions.

Liu et al. developed an injectable magnetic hydrogel incorporating Fe₃O₄ nanoparticles and an anti-inflammatory peptide (BMAP-27) to enable synergistic magnetothermal ablation and inflammation modulation. This hydrogel, designed for *in situ* application at lesion sites, responds to an externally applied alternating magnetic field (AMF), which causes localized hyperthermia (~63°C) and the thermal ablation of ectopic endometrial tissue 131. Simultaneously, magnetothermal-induced melting of the hydrogel facilitates controlled release of the BMAP-27 peptide, effectively ameliorating the pro-inflammatory microenvironment associated with endometriotic lesions. *In vivo* studies in a rat model of endometriosis showed a 90% reduction in lesion volume and significant decreases in inflammatory markers (e.g., TNF-α) without systemic toxicity or organ damage following a single injection and AMF treatment 131. Notably, the hydrogel exhibited biodegradability and retained stability, ensuring extended localized action.

While these studies highlight the versatility of tissue engineering approaches to model and treat endometriosis, several limitations and conflicting findings must be recognized. While scaffold-free bioprinting and organoid systems (e.g., Wendel et al., Zhang et al.) successfully replicate epithelial-stromal interactions and hormone responsiveness, they often lack immune and vascular components that are central to the inflammatory and angiogenic pathology of endometriosis, limiting their translational fidelity. Although microfluidic drug-screening platforms (Chen et al.) provide valuable insight into patient-specific variability, standardization remains a challenge, and their predictive accuracy for long-term therapeutic outcomes has yet to be validated in large patient cohorts. Moreover, while injectable hydrogels with drug delivery or photothermal functions (Tian et al., Liu et al.) show strong preclinical efficacy, questions remain regarding their biosafety in hormonally dynamic human environments, the reproducibility of localized heating or drug release, and the risk of off-target tissue damage. Interestingly, prevention-focused strategies, such as hydrogel-based tubal occlusion (Anthis et al.), represent a conceptual departure from lesion treatment to disease interception, but their clinical feasibility and reversibility require rigorous validation. Across these studies, methodological variability in biomaterial composition, animal models, and outcome metrics complicates direct comparison and consensus building. Importantly, most investigations remain confined to small-animal models with limited follow-up, which may not capture disease chronicity, recurrence, or the systemic immune-endocrine interplay that drives human endometriosis. Future progress will require integrative models that incorporate vascular, immune, and neural elements of the lesion microenvironment, together with large-scale comparative trials to evaluate safety, efficacy, and durability. Only through such comprehensive, cross-disciplinary efforts can tissue engineering platforms move beyond proof-of-concept systems, and evolve into clinically viable solutions for endometriosis.

### 2.4. Critical Summary and Outlook for Tissue engineering-based endometrial regeneration

Tissue engineering-based strategies for endometrial regeneration have demonstrated significant potential by integrating biomaterials, stem cells, bioactive molecules, and advanced fabrication platforms, such as 3D bioprinting and organoid systems. Compared with conventional surgical or hormonal interventions, these approaches provide superior control over structural restoration, angiogenesis, and immunomodulation, thereby enabling more physiologically relevant repair of damaged uterine tissue. Bioprinting technologies excel in spatial precision and in replicating the multilayered complexity of the endometrium, while biomaterial-stem cell composites enhance paracrine signaling, re-epithelialization, and fibrosis suppression. Moreover, organoid and organ-on-chip platforms provide unique opportunities for mechanistic investigation, disease modeling, and preclinical drug testing.

Nevertheless, limitations remain. Bioprinted constructs often face scalability and reproducibility challenges, while stem cell-based therapies raise concerns regarding sourcing, safety, and regulatory compliance. Likewise, cell-free strategies, such as extracellular vesicle delivery, demonstrate strong immunomodulatory potential, yet require rigorous standardization and optimization of dose and delivery methods. When compared across platforms, bioprinting offers unmatched architectural fidelity; biomaterial-stem cell hybrids provide potent regenerative capacity; and organoid/organ-on-chip systems contribute mechanistic insight and translational relevance.

Future progress will depend on the rational convergence of these approaches: developing hybrid systems that combine the structural precision of bioprinting, the biological efficacy of stem cells and their secretomes, and the predictive power of organoid-on-chip models. Head-to-head comparative studies and large-animal validations will be critical to determine optimal strategies for specific clinical scenarios—whether targeting fibrosis reversal, enhancing vascularization, or restoring endometrial receptivity. Ultimately, such integrative tissue engineering platforms hold the promise of advancing personalized, clinically translatable therapies for women with endometrial injury and infertility.

## 3. Recent advances in bioengineered *in vitro* platforms for endometrial tissue modeling and regeneration

### 3.1. Bioengineering endometrial organoid platforms

Organoid technology has significantly advanced the *in vitro* modeling of human endometrium, by facilitating the detailed reproduction of its dynamic hormonal responses, cellular diversity, and pathological changes with exceptional fidelity. While earlier studies using endometrial organoids cultured in Matrigel yielded important insights into epithelial biology, new bioengineering approaches are improving the physiological relevance and translational potential of these models. These improvements include the integration of organoids with custom biomaterials—from synthetic hydrogels to tissue-specific decellularized extracellular matrices—to create models that more accurately represent the native endometrial environment. Gnecco et al. engineered a synthetic ECM designed to emulate the dynamic nature of human endometrium, by fostering the co-culture of endometrial epithelial and stromal cells 132. Their method utilized a polyethylene glycol (PEG)-based hydrogel crosslinked with matrix metalloproteinase-sensitive peptides to mimic the cyclical changes of endometrium. This approach allowed hormone-induced proliferation and differentiation in epithelial organoids and stromal cells. The study showed that cells embedded in the hydrogel exhibited specific morphological and molecular responses to hormonal fluctuations 132.

Gómez-Álvarez et al. developed a novel hybrid endometrial-derived hydrogel by combining PuraMatrix (PM), a synthetic self-assembling peptide hydrogel, with porcine decellularized endometrial ECM (EndoECM) to create a biomimetic 3D microenvironment optimized for human endometrial organoid (hEO) culture and potential translational applications. This 50:50 hybrid hydrogel exhibited mechanical properties closely resembling those of native endometrial tissue, preserved key ECM components, and significantly enhanced the viability, proliferation, and differentiation of organoids into secretory and gestational phenotypes 133. Compared to standard Matrigel-based systems, the hybrid hydrogel supported higher expressions of endometrial receptivity markers such as SPP1, LIF, and PAEP and downregulated progenitor markers, indicating its functional maturity. *In vivo* implantation in immunocompetent mice confirmed the material's xenogeneic biocompatibility, stability, and minimal immune activation. These findings underscore the potential of combining organoid technology with engineered, tissue-specific biomaterials to advance personalized models and regenerative strategies in reproductive medicine.

In addition, Boretto et al. developed patient-derived organoids from endometrial diseases, such as endometriosis, hyperplasia, and cancer, that demonstrated long-term expansion and stability. These organoids replicated disease-specific features, including invasive behavior and hormone receptor expression, and accurately reflected the genomic and transcriptomic landscape of the original tissues 134. In drug screening tests, the organoids exhibited patient-specific responses, highlighting their potential for personalized medicine applications. Furthermore, when these organoids were transplanted into mice, they successfully recapitulated the original lesions, making them valuable models for studying disease progression and testing therapeutic interventions 134.

Salisbury et al. developed a tunable photo-crosslinked GelMA (gelatin methacryloyl) hydrogel system for supporting 3D cultures of primary human endometrial stromal cells and epithelial glandular organoids. Through systematic optimization of hydrogel stiffness and biochemical composition, including laminin supplementation, they discovered a GelMA formulation (10% DS100) that enhanced endometrial organoid formation and stromal cell decidualization under hormonal stimulation. Furthermore, GelMA demonstrated better mechanical stability, structural integrity over time, and compatibility with cell encapsulation and recovery than traditional matrices such as Matrigel and collagen 135. This capacity to support coculture and assembloid formation illustrates its potential as a synthetic, customizable platform for modeling complex endometrial tissue architecture and advancing reproductive biology studies *in vitro*. This research highlights the importance of engineering bioresponsive scaffolds that mimic native extracellular cues to facilitate stem cell-driven tissue reconstruction and organoid-based endometrial research.

Francés-Herrero et al. developed an advanced human endometrial organoid model by incorporating a decellularized porcine endometrium-derived ECM hydrogel (EndoECM) into the culture environment to accurately mimic the native uterine microenvironment. EndoECM supplementation significantly improved organoid proliferation, viability, and functional differentiation—as evidenced by increased Ki67 expression, glandular secretory markers, such as MUC-1 and glycogen, and epithelial progenitor cell markers like N-cadherin and SSEA-1. Notably, organoids maintained long-term chromosomal stability and successfully reproduced key histoarchitectural features of endometrial glands 136. In addition, EndoECM-supported cultures exhibited more *in vivo*-like behavior, even under nutrient-deficient conditions, than conventional Matrigel-only systems. These observations underscore the potential of combining organoid technology with tissue-specific ECM-derived biomaterials to develop physiologically relevant, stable, high-fidelity platforms for studying endometrial function, pathology, and therapeutic interventions.

Jamaluddin et al. developed hydrogel-based organoids from decellularized bovine and human endometrium, and proposed them as substitutes for Matrigel in organoid cultures and optimized decellularization methods using various detergents to preserve essential ECM components. The results obtained showed that these endometrium-derived hydrogel-based organoids effectively supported the growth of organoids from healthy and cancerous endometrial tissues. Also, organoids cultured in these hydrogels exhibited proteomic profiles that more closely resembled native endometrial tissue than those cultured in Matrigel 137. This research indicates that hydrogel-based endometrial organoids provide a more biologically relevant platform for exploring endometrial biology and its associated disorders.

Fitzgerald et al. developed self-renewing endometrial epithelial organoids (EEOs) from human uterine biopsies to model the hormonal responses and cellular composition of endometrial epithelium. They used enzymatic digestion to isolate epithelial cells from human endometrial tissue and cultured these in Matrigel under controlled conditions to form organoids. Interestingly, EEOs demonstrated hormone responsiveness, marked by alterations in the expression of genes such as FOXA2, ESR1, and PGR. These genes are crucial regulators of endometrial gland function and hormone-induced changes 138. The team utilized bulk and single-cell RNA sequencing to identify multiple epithelial cell types within the organoids, which included ciliated, unciliated, and stem cell-like populations, and observed variations in cell type proportions in response to hormone treatments.

**Figure 6:**
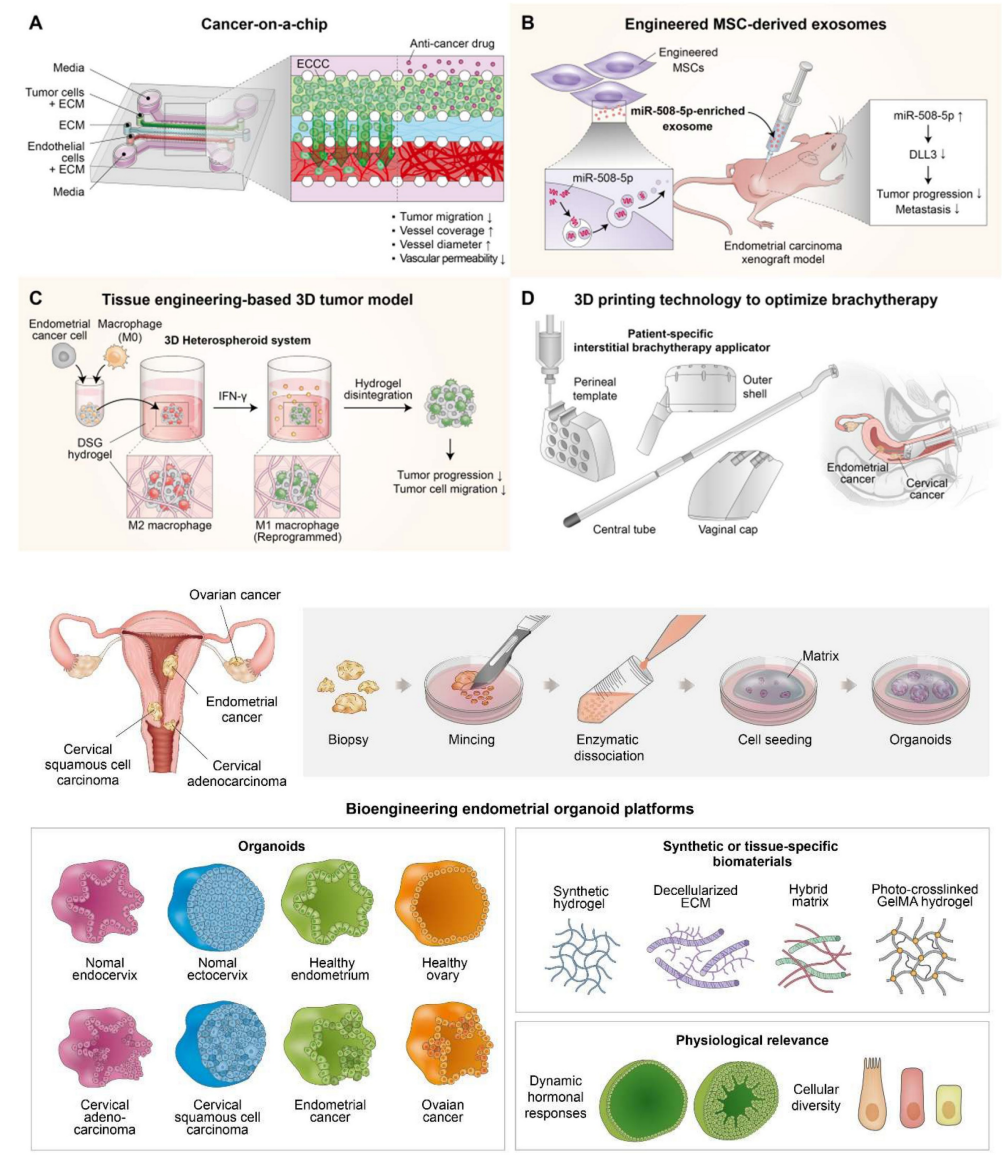
** Bioengineered organoid platforms for endometrial regeneration and modeling.** This schematic illustrates recent innovations in engineering physiologically relevant endometrial organoid systems. Traditional Matrigel-based cultures have evolved through integration with synthetic and tissue-specific biomaterials—such as PEG-based hydrogels, gelatin methacryloyl (GelMA), and decellularized extracellular matrix (ECM) derived from porcine, bovine, or human endometrium—to better replicate native uterine architecture. These platforms support hormone-responsive organoids that recapitulate epithelial diversity, stromal interactions, and disease-specific phenotypes. Functional enhancements include improved viability, differentiation, ECM remodeling, and expression of key receptivity and proliferation markers. Applications span regenerative therapies, reproductive disease modeling, and personalized drug testing. Together, these organoid-biomaterial hybrids represent a transformative tool for studying endometrial biology and advancing translational reproductive medicine.

Collectively, recent advances in bioengineered endometrial organoid platforms have facilitated the development of more physiologically and translationally relevant *in vitro* models that accurately replicate the structural, functional, and hormonal dynamics of native endometrium. By incorporating organoids with synthetic and tissue-specific biomaterials, these systems offer enhanced architectural fidelity, disease modeling accuracy, and responsiveness to therapeutic interventions (Figure 6). This approach paves the way for personalized reproductive medicine, regenerative applications, and high-throughput drug screening pursuits. Continued refinement of bioresponsive matrices and strategies involving multicellular co-cultures will undoubtedly enhance their clinical and research utility.

Despite these encouraging advances, critical challenges remain that temper the translational promise of bioengineered endometrial organoid platforms. A major limitation is the heavy reliance on Matrigel or animal-derived matrices in many early studies, which introduce variability, undefined biochemical composition, and translational incompatibility with human therapeutic use. Although synthetic alternatives, such as PEG- or GelMA-based hydrogels, provide tunability and reproducibility, they often lack the full repertoire of biochemical cues necessary to maintain long-term epithelial-stromal crosstalk, leading to discrepancies in hormonal responsiveness and differentiation outcomes across platforms. Furthermore, while disease-specific organoids (e.g., from endometriosis or cancer) replicate patient-derived genomic and phenotypic features, their ability to incorporate vascular, immune, and neural components remains limited, restricting their capacity to fully recapitulate the complex inflammatory and angiogenic milieu of the human endometrium. Notably, conflicting findings have emerged regarding the extent to which organoid-derived models preserve hormonal cycling over extended culture periods, with some reports demonstrating stable ER/PR signaling, and others observing diminished responsiveness after repeated passages. Methodological variability—including cell isolation protocols, ECM sources, and hormone supplementation strategies—further complicates cross-study comparisons and standardization. Finally, the absence of large-scale validation and the high cost of advanced biomaterial scaffolds impede scalability and clinical translation. Addressing these limitations will require integrative strategies that combine organoids with immune, vascular, and stromal co-cultures, as well as the establishment of standardized, xeno-free biomaterials to ensure reproducibility and regulatory compatibility. Only through such systematic refinement can organoid-based platforms transition from powerful experimental models to reliable clinical and translational tools in reproductive medicine.

### 3.2. Bioengineered endometrial organs-on-chips: modeling endometrial function, crosstalk, and pathology

**Figure 7:**
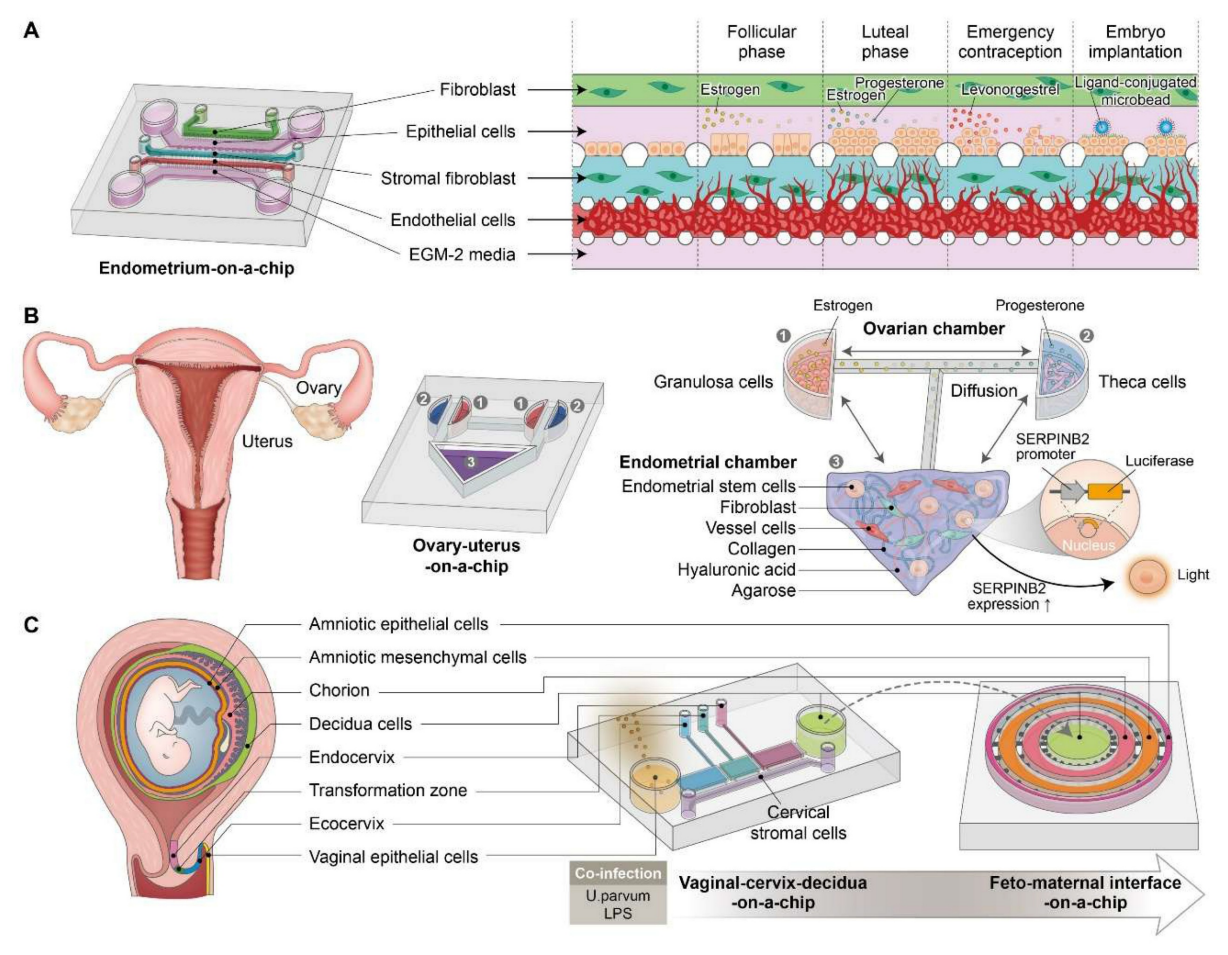
** Bioengineered endometrial organ-on-a-chip platforms for modeling function, hormonal crosstalk, and pathology.** Vascularized endometrium-on-a-chip model developed by incorporating epithelial, stromal, and endothelial cells within a microfluidic platform to replicate hormone-driven endometrial remodeling and vasculogenesis. The model responds to estradiol and levonorgestrel, enabling analysis of endometrial permeability, vascular regression, and implantation via ligand-coated microbeads **(A)**. Dual organ-on-a-chip system connecting endometrial and ovarian compartments via microchannels to facilitate bidirectional hormonal signaling. Integration of multiple uterine and follicular cell types within natural biomaterials supports cyclic hormone secretion, enhancing physiological fidelity **(B)**. VCD-FMi organ-on-a-chip platform mimicking ascending infection through the female reproductive tract. Infection with Ureaplasma parvum and LPS induces pro-inflammatory responses across anatomically compartmentalized reproductive tissues, modeling pathogen-induced obstetric complications **(C)**. Additional models include Bem et al.'s metabolic endometrium-on-a-chip simulating insulin/glucose dysregulation, Busch et al.'s microwell array for multicellular uterine architecture with hormone responsiveness, and advanced vascularized chips that support decidualization and embryo interaction. These platforms provide novel insights into hormonal response, metabolic stress, and drug responsiveness **(D)**.

Organ-on-a-chip (OOC) platforms have emerged as transformative tools in reproductive biology, by offering physiologically relevant microenvironments that accurately replicate the structural and functional complexity of human endometrium 41. By incorporating endometrial epithelial, stromal, vascular, and immune components into microfluidic systems and biomimetic scaffolds, these bioengineered models facilitate the dynamic simulation of processes such as hormone-driven remodeling, embryo implantation, metabolic modulation, and host-pathogen interactions. Recent developments in endometrium-on-a-chip technology have demonstrated the ability to mimic cyclic endocrine responses, vasculogenesis, and inter-organ communication with the ovary, in addition to modeling pathological states like inflammation and metabolic dysregulation. Furthermore, the integration of biomaterials and organoid-derived elements in these platforms enhances cellular organization, mechanical stability, and translational relevance. In this regard, Ahn et al. constructed a 3D microengineered vascularized endometrium-on-a-chip, tailored to simulate the *in vivo* structure of endometrial tissue. This model integrates epithelial cells, stromal fibroblasts, and endothelial cells into a microfluidic system, engineered to replicate physiological responses to hormonal alterations and pharmacological exposure over time. This model has successfully displayed crucial aspects of endometrial vasculogenesis and hormonal regulation, corresponding to the proliferative and secretory phases of the menstrual cycle 139. When tested with levonorgestrel, an emergency contraceptive, the model exhibited a dose-dependent increase in endometrial permeability and blood vessel regression. The platform was further validated by simulations of embryo implantation using ligand-conjugated microbeads 139 (Figure 7A provides a schematic of the 3D endometrium-on-a-chip model).

Park et al. developed a dual reproductive organ-on-a-chip model to capture bidirectional endocrine interactions between the human uterine endometrium and ovary. They constructed a platform comprised of two chambers—one for the endometrium and another for ovarian follicular cells—that enabled the exchange of hormones and cytokines through interconnected media channels. The endometrial chamber contained multiple cell types: endometrial stem cells, stromal cells, and vascular endothelial cells, integrated with biodegradable natural polymers composed of collagen, HA, or agarose. The ovarian chamber housed granulosa and theca cells 44. The study demonstrated that bidirectional endocrine communication significantly enhanced the viability and function of cells in both chambers, as evidenced by increased cell viability and hormone secretion (estrogen, progesterone, and PGE2) (a schematic of the dual reproductive organ-on-a-chip model is presented in Figure 7B).

Bem et al. used a microfluidic "endometrium-on-a-chip" model to explore the effects of varying glucose and insulin levels on endometrial function. Within the device, bovine endometrial epithelial and stromal cells were cultivated and subjected to varying glucose and insulin concentrations. Transcriptomic and proteomic analyses revealed significant alterations in gene expressions related to collagen organization, vascular development, and MAPK signaling pathways, and changes in protein abundance were observed in pathways linked to platelet activation and lysosomal activity 140. In addition, exposure to insulin influenced metabolic pathways and interactions within the ECM. These findings provide essential insights into how metabolic disruptions in the maternal environment can impact endometrial function and potentially compromise the success of early pregnancy.

Busch et al. developed a 3D microfluidic microwell array device that models the endometrial wall using patient-derived primary cells cultivated in a microfluidic platform. This model incorporates endometrial epithelial, stromal, and myometrial smooth muscle cells, which are organized into multicellular aggregates using microwell arrays 141. By refining the seeding strategies, this group determined that optimal organization occurred when smooth muscle cells were layered last, which resulted in a structured model with epithelial cells forming an exterior layer around a stromal and myometrial core. Hormonal stimulation assays validated the functionality of the model, which enabled the induction of decidualization characterized by increased IGFBP-1 and osteopontin secretion 141. Furthermore, smooth muscle cells demonstrated an increase in intracellular calcium levels in response to endothelin-1 (ET-1). This advanced model provides a significant system for investigating uterine physiology and pathologies and offers potential applications in drug testing and personalized therapeutic strategies.

Tantengco et al. developed an integrated organ-on-a-chip (OOC) system, comprised of a six-chamber vagina-cervix-decidua chip (VCD-OOC) and a feto-maternal interface chip (FMi-OOC), to model ascending infections through the female reproductive tract and examine their immunologic consequences during pregnancy. By incorporating multiple reproductive cell types into anatomically precise compartments and employing biomimetic extracellular matrices, the VCD-OOC successfully replicated epithelial-stromal interactions, collagen production, and cell-specific morphologies. Upon infection with *Ureaplasma parvum*, the system facilitated dynamic tracing of pathogen propagation, localized immune responses, and paracrine signaling to the fetal interface 142. Notably, while infection with *U. parvum* alone caused minimal inflammation, co-infection with lipopolysaccharide instigated strong pro-inflammatory cascades, simulating polymicrobial infection scenarios pertinent to preterm birth. This organoid-integrated bioengineered platform enables mechanistic studies on host-pathogen interactions within the reproductive tract and underscores the transformative potential of organ-on-chip technology in modeling complex obstetric pathologies (Summarized in Figure 7C).

Collectively, bioengineered endometrial organ-on-a-chip platforms constitute a significant advancement in reproductive research by fostering physiologically relevant, multicellular, and hormone-responsive modeling of the uterine microenvironment. These systems accurately mimic endometrial remodeling, inter-organ hormonal crosstalk, and pathological states such as metabolic dysfunction and infection. The incorporation of biomaterials and organoid technologies amplifies their translational potential for drug testing, mechanistic studies, and precision therapeutics. Future advancements in scalability and standardization are expected to further enhance their clinical and research applications.

Although organ-on-a-chip systems represent a substantial leap forward compared to traditional two-dimensional cultures, important limitations temper their translational readiness. First, the lack of standardized biomaterials and microfluidic designs complicates cross-study comparisons, as variations in scaffold stiffness, flow dynamics, and matrix composition can significantly alter hormonal responsiveness and cellular behavior. For example, while some platforms report robust decidualization and vascularization under cyclic hormone stimulation, others fail to sustain long-term hormonal sensitivity, reflecting discrepancies in biomaterial choice and chip design. Second, conflicting results have emerged regarding the capacity of these platforms to fully recapitulate embryo implantation processes: certain microfluidic models successfully simulate trophoblast invasion, whereas others demonstrate limited epithelial receptivity, raising questions about reproducibility and physiological fidelity. Third, most current devices are limited in cellular complexity—often excluding critical immune, neural, or microbiota components that are increasingly recognized as central regulators of endometrial function and pathology. Without these elements, models risk oversimplifying the multifaceted *in vivo* environment. Methodological constraints also remain, as fabrication processes can be costly, time-intensive, and difficult to scale, restricting their widespread adoption for clinical-grade applications. Finally, regulatory and translational challenges persist: unlike drug-screening assays, reproductive OOC platforms lack clear validation benchmarks and the standardized readouts required for regulatory approval. Addressing these issues will require harmonization of design parameters, incorporation of multicellular and immunologically competent components, and rigorous cross-platform validation. Only through such integrative refinements can organ-on-a-chip technologies evolve from experimental models to robust, clinically applicable platforms for diagnosing, modeling, and treating endometrial disorders.

### 3.3. Critical summary and outlook for recent advances in bioengineered *in vitro* platforms for platforms for endometrial regeneration

Recent *in vitro* bioengineered platforms—including endometrial organoids, organ-on-a-chip technologies, and hybrid 3D culture systems—have substantially advanced our ability to model human endometrial biology and regeneration. Organoids provide self-organizing multicellular structures that recapitulate hormone responsiveness and disease-specific pathophysiology, while organ-on-a-chip systems offer dynamic microfluidic environments that simulate vascularization, immune crosstalk, and cyclic remodeling under physiologically relevant conditions. Compared with traditional 2D or animal models, these *in vitro* platforms enable more accurate mechanistic studies and drug screening, with the potential to reduce translational gaps between preclinical research and clinical application.

Nevertheless, important challenges remain. Organoids lack stromal-epithelial and vascular complexity, organ-on-a-chip systems face scalability and technical standardization issues, while both approaches often rely on specialized expertise and costly equipment. Furthermore, limited consensus on culture protocols and variability in patient-derived samples hinder reproducibility across laboratories. When comparing platforms, organoids excel in recapitulating epithelial dynamics and disease modeling, while organ-on-a-chip devices more effectively capture multi-tissue interactions, mechanical forces, and temporal cyclicity.

Future progress will likely arise from integrating these complementary systems—for example, coupling organoid biology with microfluidic chips to create multi-layered, vascularized endometrial models. Such composite platforms could enable more predictive preclinical testing, personalized medicine applications, and deeper mechanistic insight into endometrial repair. To accelerate clinical translation, head-to-head comparative evaluations, standardized manufacturing protocols, and validation in large-scale reproductive models are urgently needed. Ultimately, bioengineered *in vitro* platforms hold transformative potential to bridge the gap between experimental research and therapeutic innovation in endometrial regeneration.

## 4. Emerging strategies and innovations for endometrial regeneration

### 4.1. 4D printing and endometrial regeneration

**Figure 8:**
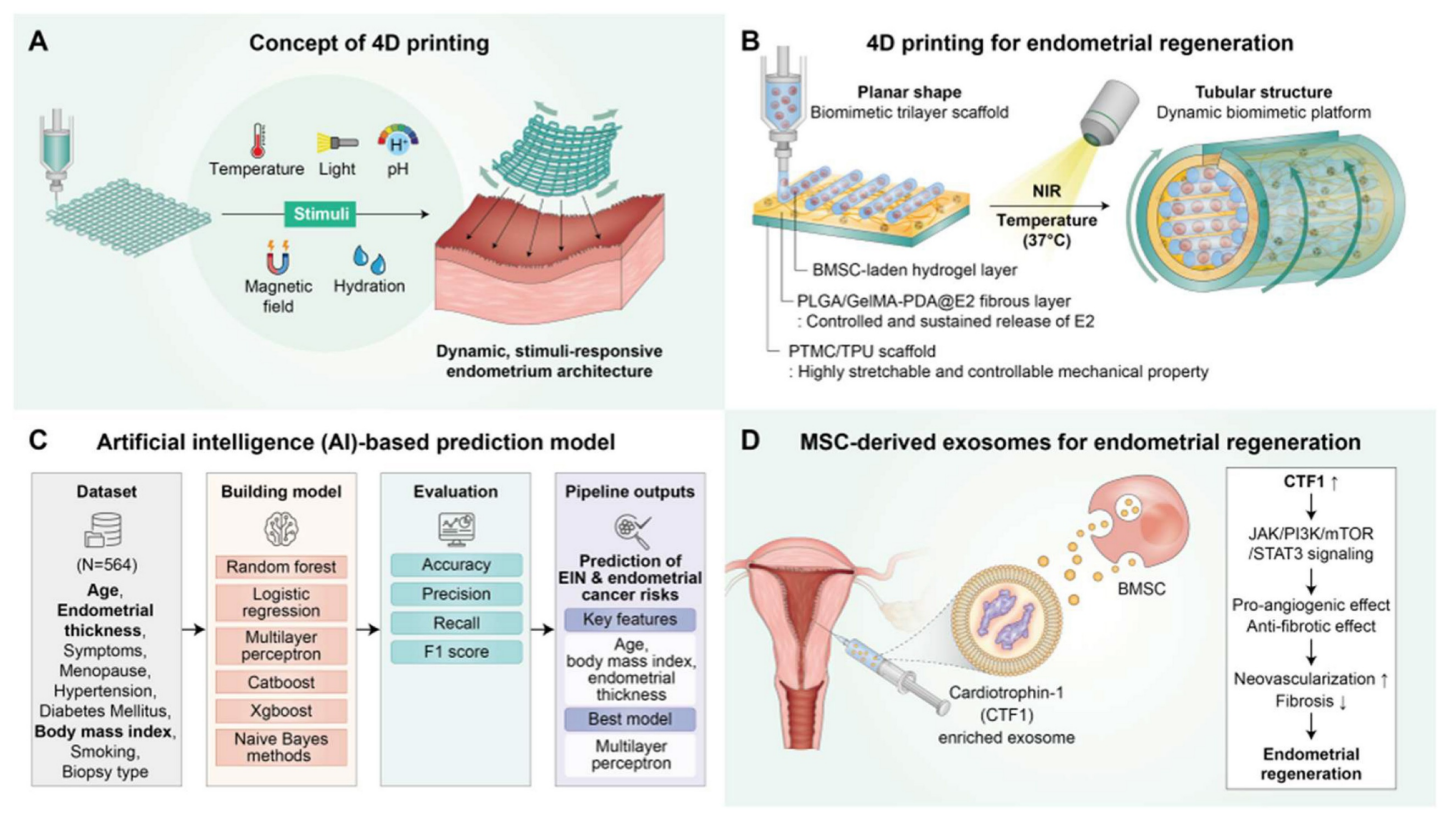
Emerging technologies in endometrial regeneration: 4D printing, artificial intelligence, and stem cell-derived extracellular vesicles. 4D bioprinting enables dynamic constructs capable of shape morphing and functional transformation in response to external stimuli such as heat, pH, or light, mimicking time-dependent tissue remodeling during regeneration (A). A shape-morphing base layer (PLLA-co-TMC/TPU) transforms into a tubular structure at body temperature, an electrospun interlayer (PLGA/GelMA) with PDA-E2 enables pH- and NIR-triggered hormone release, and a 3D-bioprinted BMSC-laden hydrogel top layer supports cell viability and differentiation. The integrated scaffold replicates uterine architecture and function (B). *AI-assisted non-invasive monitoring and diagnosis of endometrial health.* Deep learning and machine learning models enable automated assessment of endometrial thickness, histological composition, and receptivity. These models improve prediction accuracy for implantation and stratification of endometrial pathologies, supporting precision reproductive medicine (C). *Stem cell-derived extracellular vesicles (EVs) for endometrial regeneration.* BMSC- or MSC-derived EVs promote angiogenesis, reduce fibrosis, and enhance epithelial regeneration via cargo molecules such as CTF1, WWP1, miR-29a, and miR-145-5p. Mechanisms involve JAK/PI3K/mTOR, VEGF upregulation, TGF-β/SMAD inhibition, and P62-dependent autophagy (D).

4D printing technology introduces time as an active design parameter that enables the fabrication of dynamic constructs with shapes, structures, or functionalities that change in response to external stimuli such as temperature, pH, light, magnetic fields, and hydration 143. Unlike conventional 3D bioprinting, which produces static tissue constructs, 4D bioprinting enables the fabrication of dynamic, stimulus-responsive tissue architectures that can mimic the remodeling and morphogeneses of native tissues during regeneration. Two primary mechanisms—shape morphing and functional transformation—underlie these dynamic features. Shape morphing allows printed scaffolds to undergo programmable transformations from simple 2D/3D configurations into more complex 3D architectures, while functional transformation supports post-fabrication maturation and the self-organization of cells within constructs, thus facilitating tissue development controlled over time (Figure 8A).

Chen et al. developed a biomimetic trilayer scaffold by integrating 4D printing, electrospinning, and 3D bioprinting for uterine tissue regeneration with the aim of replicating the hierarchical structure and dynamic functionality of native uterine tissue 144. Utilizing 4D printing, they fabricated a shape-morphing base layer composed of PLLA-co-TMC and TPU capable of transitioning from a planar sheet to a tubular structure at body temperature and conforming to uterine curvature 144. To enhance biocompatibility and introduce therapeutic functionality, an electrospun PLGA/GelMA fibrous interlayer incorporating polydopamine (PDA) nanoparticles loaded with estradiol (E2) was added, which enabled pH-sensitive and near-infrared-triggered controlled hormone release. A final layer composed of BMSC-laden GelMA/Gel hydrogels was patterned by 3D bioprinting to provide a cell-laden microenvironment that supported cell viability, proliferation, and differentiation. The resulting scaffold demonstrated excellent mechanical properties, shape morphing capability, sustained E2 release, and *in vitro* biocompatibility, suggesting considerable potential as a dynamic, biomimetic platform for endometrial and uterine regeneration (Figure 8B).

### 4.2. AI-based technology and endometrial regeneration

The advent of artificial intelligence (AI) has ushered in a transformative era in reproductive medicine that offers novel solutions for the non-invasive assessment, diagnosis, and therapeutic monitoring of endometrial health. In the context of endometrial regeneration, AI-based technologies hold significant potential to overcome the limitations of conventional methods, which often rely on subjective clinical assessments, invasive procedures, and operator-dependent evaluations. Recent developments in AI-driven image analysis, deep learning, and machine learning algorithms enable the automated, objective, and high-throughput evaluation of key endometrial parameters, including endometrial thickness (EMT), histological composition, functional receptivity, and even oncologic risk stratification.

Mercuri et al. developed an AI-based segmentation model to efficiently and objectively measure endometrial thickness (EMT) from ultrasound images, aiming to improve clinical decision-making. Using a retrospective dataset of 70,036 sagittal-plane ultrasound images collected from 70 fertility clinics, the model was trained to automate EMT measurement based on a MaxViT-Unet architecture 145. This AI model demonstrated reliable performance, offering standardized, objective, and efficient EMT assessment, potentially reducing operator variability and improving workflow. In the context of endometrial regeneration, this technology provides a scalable, reproducible tool to monitor endometrial readiness and treatment response non-invasively, thus supporting precision medicine approaches in reproductive healthcare.

Li et al. conducted a multicenter diagnostic study developing a multimodal AI system that integrates electronic medical records (EMR) with 5,014 hysteroscopic images from 753 patients following hysteroscopic adhesiolysis for IUAs. Using a MobilenetV3-based image feature extractor and XGBoost ensemble learning, the model achieved outstanding predictive accuracy for 1-year natural conception (AUCs: 0.967 training, 0.936 validation, 0.965 test), significantly outperforming conventional IUA scoring systems and single-modal approaches 146. Importantly, the system enabled clinically actionable risk stratification: mid- to high-risk patients derived clear benefit from ART (odds ratio = 6.0, 95% CI: (1.27 to 27.8)), whereas low-risk patients demonstrated good natural conception potential without ART. This study highlights how AI-driven multimodal learning can provide precise, individualized fertility prediction and postoperative ART triage in IUA patients, thereby addressing both clinical management and cost-effectiveness challenges 146.

Li et al. recently introduced a deep learning-based prognostic system to predict fertility outcomes in patients with IUA following hysteroscopic adhesiolysis. Using a large prospective clinical database comprising 555 patients and 4,922 second-look hysteroscopic images, the authors trained transfer learning models (InceptionV3, ResNet50, InceptionResNetV2) coupled with a DeepSurv survival analysis framework 147. These AI systems achieved excellent predictive performance, with external validation AUCs of (0.94 to 0.95) for one-year pregnancy outcomes, substantially outperforming conventional scoring systems (e.g., AFS, CSGE) and clinical parameters, such as endometrial thickness. Importantly, the team developed a code-free application that stratified patients into risk groups for natural conception, and identified those most likely to benefit from ART, with a hazard ratio of 3.13 for ART benefit in patients with low natural conception probability. Visualization with Grad-CAM further enhanced interpretability by highlighting intrauterine regions most predictive of prognosis 147. Collectively, this study demonstrates that AI-driven hysteroscopic image analysis can provide accurate, objective, and clinically actionable prognostic insights, supporting personalized management and fertility counseling in IUA patients.

Lee et al. developed an AI-based deep learning model for the objective and high-throughput histological analysis of human endometrium, with focus on evaluating epithelial and stromal cellular proportions under both physiological and pathological conditions 148. Utilizing convolutional neural networks trained on digitized whole-slide images, their AI model segmented and quantified epithelial and stromal compartments with accuracies comparable to expert pathologists, overcoming conventional limitations of inter-observer variability and manual processing. Applying this model to endometrial samples from healthy women, PCOS patients, and recurrent implantation failure (RIF) patients, they demonstrated the capability of the model to detect dynamic, hormone-dependent changes in epithelial proportions throughout the menstrual cycle and to differentiate between ovulatory and anovulatory PCOS cases. Notably, the AI-based analysis revealed that anovulatory PCOS endometrium exhibited disrupted cyclical changes in epithelial composition that could contribute to impaired endometrial receptivity.

Erdemoglu et al. developed an artificial intelligence (AI)-based prediction model to assess the risk of endometrial intraepithelial neoplasia (EIN) and endometrial cancer in pre- and postmenopausal women that offered a novel approach transcending traditional symptom-based or menopausal status-dependent diagnostics 149. Using clinical parameters such as age, body mass index (BMI), and endometrial thickness—selected as key features using the Boruta algorithm—the AI model integrated multiple machine learning classifiers, such as, random forest, multilayer perceptron, and XGBoost, to achieve high diagnostic performance with an area under the curve (AUC) of 0.94. Unlike conventional diagnostic methods, which often rely on symptom recognition or invasive procedures, this AI-driven system enabled objective, non-invasive risk stratification of endometrial pathology, with focus on accessibility for underserved populations 149. Notably, the AI model demonstrated robustness at identifying high-risk individuals irrespective of menopausal status or clinical presentation, and thus, addressed healthcare disparities in endometrial cancer screening.

Fjeldstad et al. developed an AI-based model capable of non-invasively evaluating endometrial receptivity using ultrasound images to predict the likelihood of embryo implantation more accurately than conventional measures like endometrial thickness (EMT). The model integrated a deep learning (DL) component analyzing ultrasound images and a machine learning (ML) component incorporating clinical features (e.g., EMT, progesterone levels, age, prior embryo transfers) 150. Notably, EMT, though included as a key feature in the ML component, was outperformed by integrated image-based analysis in terms of predicting implantation potential. This study highlights the potential of AI-driven ultrasound analysis as a non-invasive, objective, and scalable tool for assessing endometrial receptivity and addressing the limitations of current methods, such as assessment subjectivity and unreliable receptivity assays (Figure 8C).

Despite these promising advances, significant limitations temper the clinical translation of AI-based technologies for endometrial regeneration. First, most models rely on retrospective datasets with limited diversity, raising concerns about algorithmic bias and generalizability across populations with different ethnic, demographic, or disease backgrounds. Second, while AI demonstrates high accuracy in controlled experimental or retrospective clinical settings, its reproducibility and robustness in real-world prospective trials remain underexplored. For example, models predicting endometrial receptivity often outperform traditional measures like EMT in one dataset, but when applied to external validation cohorts, show diminished accuracy, underscoring the challenge of overfitting and lack of standardized benchmarks. Third, the integration of multimodal data—including imaging, histology, hormonal profiles, and omics-based biomarkers—remains technically and computationally challenging, limiting the holistic assessment of endometrial function. Moreover, interpretability remains a major barrier: deep learning models often function as “black boxes”, which may hinder clinical trust and regulatory approval without transparent decision-making frameworks. Methodological constraints also persist in linking AI-derived predictions to mechanistic insights, as most current studies focus on outcome correlations, rather than causal understanding of regenerative processes. Finally, regulatory and ethical issues, including data privacy, standardization of digital pathology platforms, and equitable access to AI tools, remain unresolved. To advance beyond proof-of-concept studies, future work must emphasize rigorous external validation, incorporation of diverse patient cohorts, development of explainable AI frameworks, and prospective trials that directly link AI predictions to clinical outcomes. Only through such integrative refinements can AI evolve from a promising diagnostic adjunct to a reliable, clinically actionable platform to guide endometrial regeneration and fertility restoration.

### 4.3. Stem cell-derived extracellular vesicles for endometrial regeneration

Stem cell-derived extracellular vesicles (EVs), particularly exosomes, have emerged as a promising cell-free therapeutic strategy for endometrial regeneration. While mesenchymal stem cells (MSCs) have demonstrated efficacy at promoting endometrial repair through paracrine signaling, concerns regarding potential tumorigenicity, immunogenicity, and poor engraftment have limited their direct clinical application. In contrast, EVs derived from stem cells retain the regenerative bioactivity of their parent cells without associated safety risks and thus offer a scalable and biocompatible alternative for tissue regeneration. Recent studies have elucidated the multifaceted mechanisms through which MSC-derived EVs facilitate endometrial repair. In this context, Zhu et al. reported that exosomes derived from bone marrow mesenchymal stem cells (BMSCs), particularly those genetically modified to overexpress cardiotrophin-1 (CTF1), significantly enhanced endometrial regeneration and fertility restoration in a rat model of thin endometrium 151. These CTF1-overexpressing BMSC-derived exosomes (C-BMSC-exos) promoted endothelial cell proliferation, migration, and angiogenesis *in vitro*, surpassing the effects of unmodified BMSC-exos. *In vivo*, intrauterine delivery of C-BMSC-exos resulted in increased neovascularization, reduced fibrosis, and enhanced structural regeneration of endometrium and myometrium, leading to improved embryo implantation rates and higher fertility outcomes 151. Mechanistically, C-BMSC-exos activated key regenerative pathways, including the JAK/PI3K/mTOR/STAT3 pathway and thus contributed to pro-angiogenic and anti-fibrotic effects.

Zhang et al. demonstrated that EVs derived from bone marrow mesenchymal stem cells (BMSC-EVs) promote endometrial regeneration by enhancing epithelial repair, inhibiting apoptosis, and stimulating angiogenesis in injured endometrial tissue. In *in vivo* and *in vitro* models of endometrial injury, BMSC-EVs facilitated endometrial epithelial cell (EEC) proliferation, suppressed apoptosis, and indirectly promoted human umbilical vein endothelial cell (HUVEC) migration, invasion, and neovascularization 152. Mechanistically, BMSC-EVs delivered WWP1 (an E3 ubiquitin ligase), which mediated the ubiquitination of PPARγ, thereby alleviating its inhibitory effect on VEGF expression and promoting angiogenesis. Collectively, this study highlights the role of WWP1-enriched BMSC-EVs as a potent, cell-free therapeutic strategy that enhances endometrial regeneration through coordinated epithelial repair and vascular remodeling.

Tan et al. observed that exosomes derived from bone marrow mesenchymal stem cells (BMSCs-Exo) significantly promoted endometrial repair and inhibited fibrosis in a mouse model of intrauterine adhesion (IUA) 153. These BMSC-derived exosomes, characterized by classical surface markers (CD9, CD63, CD81) were effectively internalized by endometrial epithelial cells and exhibited enhance proliferation and migration *in vitro*. *In vivo*, intrauterine administration of BMSCs-Exo led to notable endometrial regeneration, characterized by increased gland formation, endometrial lining thickening, and reduced fibrotic tissue deposition 153. Mechanistic studies identified miR-29a, enriched in BMSCs-Exo, as a key anti-fibrotic factor that downregulated fibrosis-associated genes, including α-SMA, Collagen I, SMAD2, and SMAD3, thereby attenuating TGF-β/SMAD-mediated fibrogenesis during endometrial repair.

Zhou et al. reported that mesenchymal stem cell-derived exosomes (MSC-exo) effectively attenuated TGF-β1-induced endometrial fibrosis by modulating microRNA (miRNA) profiles and activating autophagy pathways 154. In their study, MSC-exo reversed the TGF-β1-mediated suppression of proliferation and enhanced apoptosis in human endometrial epithelial cells (hEECs), while also reducing the expression of fibrosis markers such as collagen I and α-SMA. Small RNA sequencing showed 15 miRNAs were differentially expressed in control and TGF-β1-induced MSC-exo-treated cells, and miR-145-5p emerged as a key regulator 154. Functional experiments showed that miR-145-5p-enriched MSC-exo alleviated fibrosis by downregulating fibrotic markers and promoting the expression of the autophagy-related protein P62. This miR-145-5p mediated the activation of P62-dependent autophagy and contributed to the anti-fibrotic and regenerative effects of MSC-exo (Figure 8D).

While stem cell-derived EVs have demonstrated remarkable regenerative potential in preclinical models, several limitations and conflicting observations warrant critical attention. First, although multiple studies report enhanced angiogenesis, reduced fibrosis, and improved fertility outcomes, the underlying molecular mediators vary widely—from protein cargo, such as WWP1 to microRNAs like miR-29a or miR-145-5p—raising questions about reproducibility and consistency across EV preparations. Differences in stem cell source, isolation methods, and culture conditions substantially influence EV composition and regenerative efficacy, complicating cross-study comparisons, while highlighting the need for standardized protocols. Second, while most studies underscore anti-fibrotic and pro-angiogenic effects, few rigorously evaluate potential off-target risks, such as excessive angiogenesis, pro-tumorigenic signaling, or long-term immune alterations, which remain major translational concerns. Third, methodological limitations persist: most studies rely on small animal models with acute endometrial injury, which may not fully recapitulate the chronic, multifactorial pathology of human IUA or thin endometrium. Moreover, the scalability and stability of EV-based therapies remain unresolved, as large-scale GMP-compliant production, cargo heterogeneity, and storage stability pose significant barriers to clinical translation. Finally, although EVs are generally regarded as safer than stem cell transplantation, the lack of regulatory consensus on whether they should be classified as biologics, drugs, or advanced therapy medicinal products creates uncertainty for clinical approval pathways. Addressing these mechanistic, methodological, and regulatory gaps will be essential to move stem cell-derived EVs from promising preclinical agents toward reliable, clinically translatable therapies for endometrial regeneration.

### 4.4. Critical summary and outlook for emerging strategies and innovations for endometrial regeneration

Emerging strategies, such as 4D bioprinting, gene- and RNA-based therapeutics, extracellular vesicle delivery, and artificial intelligence-driven platforms, represent the next frontier in endometrial regeneration. Compared with traditional biomaterial- or cell-based approaches, these innovations aim to replace damaged tissue, while also actively guiding dynamic remodeling, enhancing molecular precision, and improving predictive capabilities for clinical outcomes. 4D printing introduces stimulus-responsive constructs that can adapt to hormonal or inflammatory cues, while RNA therapeutics and exosomes offer potent means of modulating gene expression and paracrine signaling in a temporally controlled manner. In parallel, AI-enabled models are beginning to inform patient stratification and optimize treatment strategies by integrating multi-omics and clinical data.

Despite their promise, these approaches face significant hurdles. 4D printing technologies remain challenged by scalability and material standardization, while gene- and RNA-based therapies raise concerns about delivery efficiency, durability of expression, and biosafety. Similarly, exosome-based therapies require standardized isolation, characterization, and dosing protocols, and AI applications must overcome issues of data heterogeneity, algorithm transparency, and clinical validation. Cross-comparison reveals that while 4D printing excels in structural adaptability, RNA/exosome therapies provide powerful biochemical modulation, and AI contributes predictive and integrative capabilities, each platform must overcome distinct barriers before reaching clinical utility.

Looking ahead, the most impactful progress will likely come from convergence: combining adaptive biomaterial systems with RNA/exosome therapeutics, and integrating these with AI-guided modeling for personalized intervention design. To move toward translation, systematic preclinical validation, long-term safety evaluation, and harmonized regulatory frameworks will be essential. Ultimately, these emerging innovations have the potential to transform endometrial regeneration from empirical tissue replacement into a rationally engineered, precision-guided therapeutic paradigm.

## 5. Future perspectives for clinical applications

Despite significant advances in biomaterials, stem cell therapies, organoid models, and organ-on-a-chip technologies, the clinical translation of bioengineering-based strategies for endometrial regeneration faces several challenges. Bridging the gap between preclinical success and real-world therapeutic application requires not only overcoming technical and biological hurdles but also addressing regulatory, ethical, and logistical considerations. This section highlights key translational perspectives that will shape future clinical applications.

### 5.1. Overcoming regulatory and manufacturing barriers

The clinical adoption of bioengineered platforms necessitates strict adherence to Good Manufacturing Practice (GMP) standards, particularly for cell-based therapies, engineered EVs, and scaffold materials. Ensuring batch-to-batch consistency, scalability, and sterility remain critical challenges, particularly for complex constructs such as multi-layered 3D/4D printed scaffolds and patient-derived organoids. Furthermore, establishing standardized production pipelines and regulatory frameworks for stem cell-derived EVs and organoid systems is essential to facilitate their approval as advanced therapy medicinal products (ATMPs). In addition, biomaterials designed for intrauterine application must undergo rigorous biocompatibility, degradation, and toxicology testing according to ISO 10993 guidelines.

### 5.2. Personalized medicine and organ-specific therapeutic platforms

One key future direction involves leveraging patient-specific endometrial organoids and organ-on-chip models as personalized disease avatars for therapeutic screening. These platforms enable individualized drug testing and provide mechanistic insights into patient-specific responses, particularly for conditions like endometrial cancer and endometriosis. Integration of bioengineered tissues into minimally invasive delivery systems, such as injectable hydrogels or bioresorbable scaffolds, can further enhance the precision of therapeutic delivery within the uterine cavity. The combination of these personalized platforms with tissue-specific regenerative constructs holds promise for individualized treatment strategies.

### 5.3. Emerging technologies enhancing clinical translation

Next-generation technologies such as 4D bioprinting and AI-assisted diagnostic tools are expected to accelerate clinical translation. 4D bioprinting enables the creation of dynamic, stimuli-responsive implants capable of adapting to physiological changes *in vivo*, and offers significant potential for personalized, adaptive endometrial implants. Concurrently, AI-based algorithms can facilitate non-invasive, real-time monitoring of endometrial thickness, receptivity, and treatment outcomes through ultrasound or histopathological image analysis. These innovations can streamline patient selection, optimize therapeutic regimens, and enhance the monitoring of regenerative outcomes.

### 5.4. Designing clinically translatable therapies and trials

Future clinical translation strategies should prioritize the development of multifunctional, cell-free platforms, such as EV-integrated hydrogels and bioactive scaffolds, which circumvent the challenges associated with direct stem cell transplantation. Additionally, integrating regenerative biomaterials with established clinical practices, such as hysteroscopic surgery or intrauterine device placement, may facilitate regulatory approval and adoption. Designing prospective, randomized clinical trials with long-term follow-up to evaluate safety, efficacy, and fertility outcomes will be critical. Collaborations between material scientists, reproductive biologists, and clinicians are essential to ensure that preclinical innovations address unmet clinical needs.

### 5.5. Summary of clinical translation barriers for tissue engineering products in endometrial regeneration

While tissue engineering has achieved remarkable preclinical progress in restoring endometrial structure and function, the path toward clinical translation remains fraught with obstacles. The transition from laboratory innovation to clinically applicable therapies requires not just proof of regenerative efficacy, but also rigorous consideration of manufacturing feasibility, regulatory compliance, surgical applicability, and economic sustainability. A critical appraisal of these barriers is therefore essential to understand why many promising biomaterial- and bioprinting-based strategies have yet to achieve widespread clinical adoption, and to identify the key priorities that must be addressed for the field to progress.

Despite the extensive preclinical promise of natural and synthetic biomaterials-based tissue engineering products, several barriers continue to hinder their seamless clinical translation. First, the biological variability of source tissues (e.g., donor age, sex, tissue type, and processing conditions) leads to inconsistency in scaffold properties and regenerative outcomes, complicating reproducibility across studies and products. Second, processing and manufacturing challenges—including decellularization, sterilization, and large-scale GMP-standard production—affect the preservation of bioactivity while ensuring mechanical integrity and safety, and the lack of universal quality standards exacerbates batch-to-batch variability. Third, surgical implantation techniques significantly influence clinical outcomes, with discrepancies in placement methods (e.g., onlay *vs.* sublay) contributing to recurrence and inconsistent performance. Fourth, regulatory hurdles remain substantial, as ECM biomaterials occupy a grey zone between medical devices and biologics. While some products follow streamlined device approval (510k), others require cost- and time-intensive biologics license applications (BLA) or tissue-engineered product classifications, leading to fragmented approval pathways across the FDA, EMA, and other agencies. Finally, economic limitations pose another critical barrier: ECM scaffolds are markedly more expensive than synthetic alternatives, and despite potential long-term benefits, high upfront costs and uncertain reimbursement policies restrict their widespread adoption. Collectively, these barriers underscore the need for standardized manufacturing protocols, harmonized regulatory frameworks, cost-effectiveness analyses, and large-scale randomized clinical trials. Addressing these challenges will be pivotal to transforming biomaterial-based therapies from promising experimental tools into reliable, clinically translatable solutions for endometrial regeneration 155.

Although 3D bioprinting has emerged as a transformative tool in tissue engineering, several critical barriers continue to impede its clinical translation. Bioink limitations remain central: natural hydrogels (e.g., collagen, gelatin, alginate) often suffer from poor mechanical strength, batch-to-batch variability, and animal-origin immunogenicity, whereas synthetic bioinks provide reproducibility but lack essential bioactivity, highlighting the absence of a universally accepted “tissue-specific” bioink standard. Scalability and reproducibility also pose major challenges—bioprinted constructs frequently fail to achieve clinically relevant size and function, due to long printing times, inadequate vascularization, and limited cell survival during large-scale fabrication. Moreover, biomechanical integration remains a bottleneck, as most printed constructs lack the durability and functional maturity required to withstand physiological loading. Coupled with this, cell sourcing and maturation present significant hurdles: the large numbers of autologous or stem cells required for clinical-scale constructs are difficult to obtain, expand, and differentiate safely. On the translational front, regulatory and ethical uncertainties are substantial. Bioprinted tissues and organs often fall into “combination product” categories that blur the lines between biologics, medical devices, and advanced therapy medicinal products, resulting in fragmented approval pathways across global agencies. This regulatory ambiguity, combined with high manufacturing costs and limited GMP-compliant standards, continues to slow commercialization 156.

In summary, overcoming the barriers to clinical translation will require a multipronged strategy that integrates standardized, GMP-compliant manufacturing protocols, biologically and mechanically optimized bioinks and scaffolds, and robust vascularization and maturation strategies to ensure functional long-term integration. Equally important will be the establishment of harmonized international regulatory frameworks and cost-effectiveness analyses to facilitate commercialization and patient access. By systematically addressing these scientific, technical, regulatory, and economic challenges, tissue engineering approaches for endometrial regeneration can evolve from promising experimental prototypes into transformative clinical therapies that are able to restore fertility and reproductive health.

## 6. Conclusion

Endometrial regeneration represents a frontier in reproductive medicine, characterized by significant advances in biomaterials, stem cell therapies, 3D bioprinting, and organ-on-a-chip technologies. These approaches have demonstrated the ability to mimic the intricate endometrial microenvironment, promote tissue repair, and enable functional restoration. Both natural and synthetic polymers, combined with stem cells, have remarkably enhanced fertility outcomes by reconstructing native uterine tissue. Stem cell-based organoid platforms have provided valuable tools for elucidating disease mechanisms and offer the prospect of personalized therapeutic interventions. Meanwhile, organ-on-a-chip technologies are enhancing our ability to model endometrial responses to metabolic and hormonal changes, and thus facilitate more precise drug testing and mechanistic studies. Despite these advancements, numerous challenges persist. Achieving complete integration of engineered tissues with native endometrium, ensuring long-term functionality, and preventing immune rejection are essential areas for further research. In addition, the translation from preclinical models to human applications requires comprehensive clinical trials to confirm safety and efficacy. Future studies should focus on optimizing the scalability and reproducibility of these technologies for clinical applications, and on developing personalized, tissue-engineered constructs tailored to the needs of individual patients. The integration of multidisciplinary approaches, involving expertise from materials science, biology, and reproductive medicine, is crucial for addressing current limitations. Ongoing research in this domain has the potential to revolutionize treatments for endometrial disorders and provides new hope for patients facing infertility and associated reproductive challenges. In summary, the convergence of biomaterials science and cutting-edge bioengineering technologies, ranging from smart biomaterial scaffolds and 4D bioprinting to AI-assisted diagnostics and extracellular vesicle therapeutics, presents a clinically promising framework for endometrial regeneration and fertility restoration. While biomaterial-based platforms demonstrate strong regenerative efficacy in preclinical models, successful clinical translation will depend on addressing critical challenges related to scalable manufacture, regulatory approval, and long-term safety validation.
